# Transgressive hybrids as hopeful holobionts

**DOI:** 10.1186/s40168-024-01994-8

**Published:** 2025-01-22

**Authors:** Benjamin Thomas Camper, Andrew Stephen Kanes, Zachary Tyler Laughlin, Riley Tate Manuel, Sharon Anne Bewick

**Affiliations:** 1 Department of Biological Sciences, Clemson University, Clemson, SC 29631 USA; 2 Department of Ecology and Evolutionary Biology, University of Michigan, Ann Arbor, MI 48109 USA

**Keywords:** Hybridization, Holobiont, Parthenogen, *Aspidoscelis*, Microbiome, Reticulate evolution, Hopeful monster, Transgressive segregation, 16S

## Abstract

**Background:**

Hybridization between evolutionary lineages has profound impacts on the fitness and ecology of hybrid progeny. In extreme cases, the effects of hybridization can transcend ecological timescales by introducing trait novelty upon which evolution can act. Indeed, hybridization can even have macroevolutionary consequences, for example, as a driver of adaptive radiations and evolutionary innovations. Accordingly, hybridization is now recognized as a motor for macrobial evolution. By contrast, there has been substantially less progress made towards understanding the positive eco-evolutionary consequences of hybridization on holobionts. Rather, the emerging paradigm in holobiont literature is that hybridization disrupts symbiosis between a host lineage and its microbiome, leaving hybrids at a fitness deficit. These conclusions, however, have been drawn based on results from predominantly low-fitness hybrid organisms. Studying “dead-end” hybrids all but guarantees finding that hybridization is detrimental. This is the pitfall that Dobzhansky fell into over 80 years ago when he used hybrid sterility and inviability to conclude that hybridization hinders evolution. Goldschmidt, however, argued that rare saltational successes—so-called hopeful monsters—disproportionately drive positive evolutionary outcomes. Goldschmidt’s view is now becoming a widely accepted explanation for the prevalence of historical hybridization in extant macrobial lineages. Aligning holobiont research with this broader evolutionary perspective requires recognizing the importance of similar patterns in host–microbiome systems. That is, rare and successful “hopeful holobionts” (i.e., hopeful monsters at the holobiont scale) might be disproportionately responsible for holobiont evolution. If true, then it is these successful systems that we should be studying to assess impacts of hybridization on the macroevolutionary trajectories of host–microbiome symbioses.

**Results:**

In this paper, we explore the effects of hybridization on the gut (cloacal) and skin microbiota in an ecologically successful hybrid lizard, *Aspidoscelis neomexicanus*. Specifically, we test the hypothesis that hybrid lizards have host-associated (HA) microbiota traits strongly differentiated from their progenitor species. Across numerous hybrid microbiota phenotypes, we find widespread evidence of transgressive segregation. Further, microbiota restructuring broadly correlates with niche restructuring during hybridization. This suggests a relationship between HA microbiota traits and ecological success.

**Conclusion:**

Transgressive segregation of HA microbiota traits is not only limited to hybrids at a fitness deficit but also occurs in ecologically successful hybrids. This suggests that hybridization may be a mechanism for generating novel and potentially beneficial holobiont phenotypes. Supporting such a conclusion, the correlations that we find between hybrid microbiota and the hybrid niche indicate that hybridization might change host microbiota in ways that promote a shift or an expansion in host niche space. If true, hybrid microbiota restructuring may underly ecological release from progenitors. This, in turn, could drive evolutionary diversification. Using our system as an example, we elaborate on the evolutionary implications of host hybridization within the context of holobiont theory and then outline the next steps for understanding the role of hybridization in holobiont research.

Video Abstract

**Supplementary Information:**

The online version contains supplementary material available at 10.1186/s40168-024-01994-8.

## Introduction

Hybridization in macrobial systems facilitates evolution through lineage reticulation (i.e., unification), typically via unidirectional introgression or bidirectional admixture. This contrasts with more traditional mechanisms of evolution involving lineage diversification. Whereas genomic change due to divergent processes tends to be incremental, hybridization yields immediate, extensive genomic change. For this reason, hybrid systems are often cradles of phenotypic novelty [1, 2] and evolutionary innovation [3–6]. In the past, hybrid systems have been studied as generators of phenotypic novelty in behavior, [7, 8] fecundity [9–13], morphology [14–17], and physiology [18–22]. Recently, however, there has been growing recognition that many ecologically relevant organismal traits are not determined by an organism’s genes alone. Instead, macroorganisms function as “holobionts”—collectives comprising both a host organism and all of its host-associated (HA) microbes [23–25]. As a consequence, many host traits are jointly or even solely governed by the actions of HA microbes [26–28]. How host hybridization impacts the HA microbiome, the extent to which these changes influence other host traits, and the downstream evolutionary consequences for the holobiont remain open questions.

To the extent that host hybridization has been studied within holobiont literature, host hybridization has been viewed as a disruptive force. Indeed, the existing paradigm is that host hybridization leads to host–microbiome mismatches and breakdowns of mutualistic relationships [29–32]. As a consequence, most efforts to characterize interactions between HA microbiomes and host evolution [29] have focused on host–microbiome relationships from the perspective of host lineage diversification. This narrow focus of holobiont research on lineage diversification is, however, noticeably at odds with broader evolutionary perspectives that increasingly accept hybridization as an important mode of evolution [33]. Indeed, in macrobial systems, hybridization is now considered something of a double-edged sword. In many cases, hybridization serves as a brake, slowing the rate of evolutionary change. But in other cases, it is actually a motor, promoting host lineage diversification [34–36]. In the systems where hybridization is beneficial, success is often attributed to transgressive segregation (i.e., extreme or novel phenotypes) [21, 37, 38]. However, despite evidence for transgressive segregation of microbial phenotypes, very little effort has been invested in testing the hypothesis that hybridization can generate beneficial novelty in holobiont traits.

The potential for host hybridization to positively impact holobiont evolution has likely been underestimated because the majority of holobiont hybridization studies have considered hybrids at a fitness deficit [31, 39, 40]. This is most obvious in several hallmark studies linking transgressive segregation of the hybrid microbiota to fitness deficits in *Nasonia* wasps [39] and *Mus musculus* mice. [40] Unfortunately, focusing on “dead-end” hybrids strongly biases results towards finding the negative impacts of host hybridization on host–microbiome symbioses (but see Malukiewicz et al. [41] and Zhu et al. [42] for studies where hybrids were not at an obvious fitness deficit). However, while holobiont fitness deficits may be the most likely outcomes of host hybridization, dead-end hybrids are not the most fruitful for understanding the positive evolutionary consequences of hybridization. Notably, associating prevalence with significance was the mistake made by Dobzhansky who, during early hybridization research, argued that hybridization hinders macrobial evolution based on the fact that hybrid organisms are frequently sterile and/or inviable [11, 43–46]. Goldschmidt, on the other hand, avoided this logical fallacy by recognizing that rare positive outcomes—so-called hopeful monsters—could disproportionately drive evolutionary diversification [47, 48]. Although Goldschmidt’s arguments were formulated in a broader “macromutation” context, the pattern that he described has since been extensively applied to hybridization. In particular, recent authors have outlined how genetic restructuring through hybridization serves as a common mechanism for generating macromutations [49–54] that can drive evolutionary diversification in macrobial systems [5, 35, 55–60].

Since Goldschmidt, appreciation for the role of hybridization has accelerated, driven in part by early adopters like Anderson [61, 62] and Stebbins, [63, 64] as well as the prevalence of DNA sequencing techniques in the field of evolution [65]. Thus, hybridization is now understood as a common and important mode of evolution. Indeed, many successful clades show widespread evidence of historical introgression, and in some taxa, hybridization has even been associated with increased diversification rates, such as adaptive radiations [5, 35, 55–57, 65, 66]. These studies have led to a paradigm shift from modeling clade evolution as a branching tree focused on lineage diversification to an evolutionary network comprising both lineage divergence and reticulation [67]. Aligning holobiont research with this broader evolutionary perspective requires recognizing the importance of similar patterns in host–microbiome systems. In other words, like Goldschmidt’s hopeful monsters, rare, successful “hopeful holobionts” might be disproportionately responsible for holobiont evolution, even when most hybrid holobionts suffer fitness deficits (e.g., disruptions to host–microbe symbioses). If this hypothesis is true, then the impact of holobiont trait novelty on evolutionary success will likely only be apparent by focusing on successful hybrid systems.

Whiptail lizards (genus = *Aspidoscelis*) of North America serve as unique examples of ecologically successful hybrid vertebrates. Among *Aspidoscelis*, there are a total of eight diploid and seven triploid parthenogenetic species [68–70] (although these numbers may change based on new discoveries and species definitions [71–73]), all of which spawned as a result of natural hybridization between different progenitor species, and many of which exhibit ecological shifts [74–76] or expansion [77–80] into novel habitats and locations. Beyond serving as examples of successful hybridization, whiptail lizards have several other advantages. Unlike many traditional hybrid systems that backcross with their progenitor populations, parthenogenetic whiptails emerge through single hybridization events that give rise to independently reproducing clonal lineages [80, 81]. Thus, populations (termed “arrays” [72, 82] though we will refer to them as “populations” to avoid confusion) of hybrid parthenogens maintain very little interindividual genetic variation and effectively represent the “frozen” F_1_ generation of a hybrid cross. This means that, unlike introgression zones, hybrid parthenogens have a fixed genetic composition that is almost fully intermediate (i.e., 50% maternal progenitor genome and 50% paternal progenitor genome) to either progenitor species. Further, because of their intermediate genetic status, hybrid parthenogens exhibit maximum heterozygosity at alleles that are differentially fixed in progenitor populations [83]. Additionally, because parthenogens resemble F_1_ generations, the “front-end” effects of hybridization are effectively preserved. This permits better study of the effects of early hybridization than most other types of hybrid systems. A final and important advantage of whiptails is that many parthenogenetic *Aspidoscelis* species occur syntopically or, at least, sympatrically with one or both progenitor species [78, 79, 84–86]. As a result, *Aspidoscelis* systems are excellent natural laboratories for comparing traits, including microbial phenotypes, between progenitor species and their hybrid offspring while controlling for the effects of habitat variation. Despite these many advantages, few published studies have addressed the effects of hybridization on HA microbiomes of parthenogenetic whiptails or even hybrid vertebrate parthenogens in general.

In this paper, we use the ecologically successful diploid hybrid parthenogen, *Aspidoscelis neomexicanus*, to test the hypothesis that successful hybrid holobionts exhibit HA microbiota traits that are differentiated from those of their progenitors. Finding that most *A. neomexicanus* HA microbiota traits are, in fact, distinct from their progenitors, we further test the hypothesis that successful hybrid holobionts exhibit HA microbiota traits that are transgressive to their progenitors. We expect *A. neomexicanus* HA microbiota traits to be transgressive rather than intermediate for several reasons. First, *A. neomexicanus* exhibits some transgressive ecological traits [79, 87, 88]. Thus, to the extent that HA microbiota traits either cause or reflect an altered ecological niche, the HA microbiota traits of *A. neomexicanus* should be transgressive. Second, existing data from macrobial systems suggest that transgressive phenotypes are more common in hybrids, like *A. neomexicanus*, whose progenitor species are distantly related [89, 90]. To test our hypotheses, we sampled both the skin (cutaneous) and cloacal (representative of “gut”) microbiota of *A. neomexicanus*, where it occurs syntopically with each of its progenitor species, *Aspidoscelis marmoratus* (maternal) and *Aspidoscelis inornatus* (paternal) [68]. We then considered a range of different HA microbiota traits, including both diversity and composition measures. For each trait, we classified *A. neomexicanus* as transgressive (novel), intermediate (between each progenitor), or conserved (resembling one or both progenitors) relative to its two progenitor species.

Our findings suggest that most HA microbiota traits in *A. neomexicanus* are indeed distinct from and transgressive to the traits of both progenitor species. By relating observed differences between hybrid and progenitor microbiota to known differences in ecological niche, we use these results to speculate on potential mechanisms by which hybridization produces phenotypic novelty in holobionts and the possible ecological consequences of such novelty. More broadly, we use our results to discuss the largely overlooked potential for ecological differences that emerge in hybrid holobionts to influence holobiont macroevolution. Consistent with early evolutionary theory, holobiont evolutionary literature has adopted a paradigm wherein hybridization is viewed as a primarily negative phenomenon. By shifting focus to successful hybrid systems, we aim to align holobiont research with modern evolutionary theory that recognizes hybridization as an important mode of evolution.

## Results

Throughout the “Results” section, we apply phylogenetically aware incidence-based metrics (i.e., Faith’s phylogenetic diversity [91] and unweighted UniFrac [92]) to amplicon sequence variants (ASVs, i.e., sequences that differ by at least one base pair, regardless of taxonomic assignment) to characterize microbiota structure and composition. We choose an incidence-based approach because unlike an abundance-based approach, it can be applied to all of the HA microbiota traits that we consider, including traits that are calculated across multiple lizards (e.g., *β*-diversity, *γ*-diversity). We choose a phylogenetically aware approach because it captures differentiation in a less arbitrary manner than using a specific taxonomic level (e.g., species, genera) or similarity threshold (e.g., 97% similar operational taxonomic units). However, we also provide numerous abundance-based and phylogenetically agnostic measures, including genus-level analyses, in the additional files. As expected, most of our results are consistent across metrics, though there are occasional differences. For all analyses, we consider two sites—“Sevilleta Northwest,” (SNW) where *A. marmoratus* (maternal progenitor) occurs syntopically with *A. neomexicanus*, and “Sevilleta Blue Grama” (SBluG), where *A. inornatus* (paternal progenitor) occurs syntopically with *A. neomexicanus*. Throughout the remainder of the manuscript, we refer to a “population” as a single lizard species from a single study location (e.g., *A. neomexicanus* at SBluG).

To test our hypotheses that hybrid microbiota traits are (1) distinct and (2) transgressive in hybrid animals, we compare hybrid and progenitor HA microbiota as follows:

- If a hybrid microbiota trait is significantly different from and more extreme than both progenitors (e.g., a more extreme value for diversity, value along a PCoA axis, or value of microbial abundance), that hybrid microbiota trait is transgressive.
- If a hybrid microbiota trait is significantly different from both progenitors but lies between the two progenitor values (e.g., an intermediate value for diversity, value along a PCoA axis, or value of microbial abundance), that hybrid microbiota trait is intermediate.
- If a hybrid microbiota trait is not significantly different from one or both progenitors (e.g., the same diversity, PCoA axis position, or microbial abundance), that hybrid microbiota trait is conserved.

Table 1 summarizes our results across all holobiont traits and both body sites. To construct this table, we consider each trait and each hybrid population (SBluG and SNW) separately. We then assess whether that particular hybrid population exhibits a transgressive, conserved, or intermediate trait value compared to both progenitor populations (see Table 2 for specific hybrid trait outcomes). This allows us to tally the total support for trait differentiation and transgressive segregation across both populations and all traits. Overall, for both gut and skin microbiota, we observe more trait differentiation than trait conservation (Hypothesis 1) and more transgressive segregation than intermediacy (Hypothesis 2). This is particularly apparent for diversity but is also true for microbial abundances. By contrast, we observe more support for trait conservation in our composition analyses. Comparing gut and skin microbiota, we observe more support for trait differentiation in the skin; however, we observe more support for transgressive segregation in the gut. In what follows, we discuss each trait individually and in more detail.

**Table 1:** Number of trait × hybrid population measures supporting each of our three (transgressive, intermediate, or conserved) possible outcomes. For both gut and skin microbiota, we consider 7 diversity traits in each of our 2 hybrid populations (i.e., 14 traits × hybrid population measures), 4 composition traits in each of our 2 hybrid populations (i.e., 8 traits × hybrid population measures), and 2 microbial abundance traits in each of our 2 hybrid populations (i.e., 4 traits × hybrid population measures). Percentages of trait × hybrid population measures for each outcome for each category (i.e., diversity, composition, and microbial abundance) are shown in parentheses

	Gut			Skin		
	Transgressive	Conserved	Intermediate	Transgressive	Conserved	Intermediate
Diversity	8 (57.14%)	6 (42.86%)	0 (0%)	7 (50%)	5 (35.71%)	2 (14.29%)
Composition	2 (25%)	5 (62.50%)	1 (12.50%)	3 (37.50%)	3 (37.50%)	2 (25%)
Abundance	4 (100%)	0 (0%)	0 (0%)	2 (50%)	2 (50%)	0 (0%)
Total	14 (53.85%)	11 (42.30%)	1 (3.85%)	12 (46.15%)	10 (38.47%)	4 (15.38%)

**Table 2:** Summary of results for the various hybrid microbiota traits that we consider

	**Both progenitors**		**Syntopic progenitor**	
	**Gut**	**Skin**	**Gut**	**Skin**
**Diversity**				
* α*-diversity	**C**^**M+I∧**^ **C**^**M+I**^^∧ ^*	T^∧^ C^M+I^^∧ ^*	**C**^**∧**^ **C**^∧ ^*	D^∧^ C^∧ ^*
* β*-diversity	T_∨_ C^M+I^_∨ _*	C^M^- T_∨_	D_∨_ C_∨ _*	**D**_**∨**_** D**_∨ _*
* γ*-diversity	**T**^**∧**^ **T**^∧ ^*	T^∧^ I	**D**^**∧**^ **D**^∧ ^*	**D**^**∧**^ **D**^∧ ^*
Core diversity (absolute)	C^M^^∧^ T^∧ ^*	**T**^**∧**^ **T**^∧ ^*	**D**^**∧**^** D**^∧ ^*	**D**^**∧**^ **D**^∧ ^*
Core diversity (%)	**C**^**M+I∧**^ **C**^**M**^^∧ ^*	**C**^**M**^**-** **C**^**M**^^**∧**^	**C**^**∧**^ **C**^∧ ^*	D^∧^ C^∧ ^*
Unique core diversity (absolute)	**T**^**∧**^ **T**^∧ ^*	T^∧^ C^I^^∧ ^*	**D**^**∧**^ **D**^∧ ^*	**D**^**∧**^ **D**^∧ ^*
Unique core diversity (%)	**T**^**∧**^** T**^∧ ^*	T^∧^ I	**D**^**∧**^ **D**^∧ ^*	**D**^**∧**^** D**^∧ ^*
**Composition**				
PCoA axis 1	**C**^**M+I**^_∨_ **C**^**M**^_∨ _*	T^∧^ C^M+I∧ ^*	**C**_**∨**_** C**_∨ _*	D^∧^ C^∧ ^*
PCoA axis 2	**C**^**I∧**^ **C**^**M+I**^^**-**^	**C**^**I**^**- C**^**M∧**^	**C**^**∧**^ **C**^∧ ^*	**C**^**∧**^** C**^∧ ^*
20D axis of parental variation	I C^M^_∨_	**I I**	D^∧^ C_∨_	**D**^**∧**^ **D**_∨ _*
20D height of triangle	**T**^**∧**^** T**^∧ ^*	**T**^**∧**^** T**^∧ ^*	**D**^**∧**^ **D**^∧ ^*	**D**^**∧**^ **D**^∧ ^*
**Microbial abundance**				
* Dietzia maris*	**T**_**∨**_ **T**_∨ _*	**T**_**∨**_ **T**_∨ _*	**D**_**∨**_** D**_∨ _*	**D**_**∨**_ **D**_∨ _*
* Corynebacterium testudinoris*	**T**_**∨**_ **T**_∨ _*	---	**D**_**∨**_ **D**_∨ _*	---
* Fodinibacter luteus*	---	**C**^**I**^_**0**_** C**^**M**^**-**	**---**	**C**_**0**_** C**_**∨**_

When comparing a hybrid population to both progenitor populations, a trait can be transgressive (T), intermediate (I), or conserved (C) in the hybrid. When a trait is conserved, it can be similar to *A. inornatus* (^I^), similar to *A. marmoratus* (^M^), or similar to both progenitors (^I+M^). Further, when a trait is conserved, the median (diversity or microbial abundance) or mean (composition) value of that trait can be higher (^∧^) or lower (_∨_) than the median/mean value of both progenitors. Alternatively, it can be intermediate (_-_) to the median/mean values of the two progenitors. Likewise, when a trait is transgressive, it can be higher (^∧^) or lower than both progenitors (_∨_). When comparing a hybrid population to its syntopic progenitor population, a trait can be differentiated (D) or conserved (C) in the hybrid. In both cases, the median/mean value of the trait can be higher (^∧^) or lower (_∨_) than the median/mean value of its syntopic progenitor or can be identical (0) to its syntopic progenitor. The first letter in each column refers to the hybrid population at SBluG. The second letter in each column refers to the hybrid population at SNW. Traits with consistent outcomes (C/T/I or D/C) across hybrid populations are indicated in bold. Traits with similar trends across hybrid populations (independent of significance) are indicated by an asterisk (*)

### Microbiota diversity

We consider seven microbiota diversity measures. These are as follows: *α*-diversity (i.e., diversity on a single animal), *β*-diversity (i.e., variation between animals), *γ*-diversity (i.e., diversity on an entire population), core diversity (i.e., diversity of the portion of the microbiota that is conserved across an entire population), percent core diversity (i.e., percentage of *γ*-diversity that is part of the core), unique core diversity (i.e., diversity of the portion of the core not shared with the core of any other population), and percent unique core diversity (i.e., percentage of a population’s core diversity that is unique).

#### α-Diversity

**Fig. 1:**
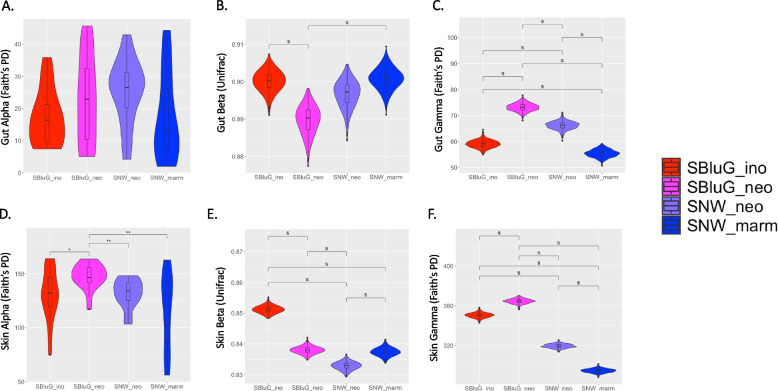
Comparison of diversity between populations of *A. inornatus* from SBluG (red), *A. neomexicanus* from SBluG (magenta), *A. neomexicanus* from SNW (purple), and *A. marmoratus* from SNW (blue) for both gut (**A**, **B**, **C**) and skin (**D**, **E**, **F**) microbiota (ASVs) and considering *α*-diversity (**A**, **D**), *β*-diversity (**B**, **E**), and *γ*-diversity (**C**, **F**). **A** and **D** are based on the microbiota of individual lizards from each population; **B**, **C**, **E**, and **F** are based on 500 bootstraps of 15 lizards from each population using the methods outlined in Chao et al. [93, 94] Significant differences in *α*-diversity between groups, as determined by a Kruskal–Wallis test followed by post hoc pairwise Wilcox tests using a Benjamini–Hochberg correction, are indicated as follows: *p*-value ≤ 0.001 (***), *p*-value ≤ 0.01 (**), *p*-value ≤ 0.05 (*), and *p*-value ≤ 0.1 (.) Significant differences in *β*- and *γ*-diversity, as determined by overlap of 83.4% confidence intervals, are indicated with an “s.” Additional analyses using alternate diversity metrics can be found in Additional file 1

The majority of pairwise comparisons between single hybrid populations and single progenitor populations do not indicate significant differences in gut or skin *α*-diversity (see Fig. 1A, D; see Additional file 1: Figs. 1.1, 1.2, 1.3, 1.4, 1.5, 1.6 for additional metrics). The sole exception is *α*-diversity of the skin microbiota of hybrid lizards at SBluG, which is significantly higher than *α*-diversity of the skin microbiota of either progenitor population. While *α*-diversity of hybrid populations is not significantly different from progenitor populations across most pairwise comparisons, median *α*-diversity of all hybrid populations is higher than median *α*-diversity of all progenitor populations. Further, combining the two hybrid populations into a single “asexual” class and the two progenitor populations into a single “bisexual” class results in significantly greater hybrid *α*-diversity (see Additional file 1: Figs. 1.2 and 1.4).

#### β-Diversity

The majority of pairwise comparisons between single hybrid populations and single progenitor populations indicate that *β*-diversity is significantly lower in hybrid populations (see Fig. 1B, E; see Additional file 1: Figs. 1.7, 1.8 for additional metrics). The exceptions are *β*-diversity of the gut microbiota of hybrid lizards at SNW, which is not significantly different from *β*-diversity of the gut microbiota of either progenitor population, and *β*-diversity of the skin microbiota of hybrid lizards at SBluG, which is not significantly different from *β*-diversity of the skin microbiota of progenitor lizards at SNW. Despite lack of statistical significance, median *β*-diversity of the gut microbiota of hybrid lizards at SNW is lower than median *β*-diversity of both progenitor populations. This is not the case, however, for median *β*-diversity of the skin microbiota of hybrid lizards at SBluG, which is actually higher than median *β*-diversity of the skin microbiota of progenitor lizards at SNW. Indeed, this is the sole comparison where median *β*-diversity of a hybrid population is higher than median *β*-diversity of a progenitor population. This result, however, warrants further discussion. In particular, it is important to note that median *β*-diversity of the skin microbiota of both populations at SBluG is higher than median *β*-diversity of the skin microbiota of both populations at SNW. Further, *β*-diversity of the skin microbiota of progenitor lizards at SBluG is significantly higher than *β*-diversity of the skin microbiota of progenitor lizards at SNW, and *β*-diversity of the skin microbiota of hybrid lizards at SBluG is significantly higher than *β*-diversity of the skin microbiota of hybrid lizards at SNW. Thus, factors at the SBluG location appear to inflate *β*-diversity of the skin microbiota, potentially obscuring differences due to lizard species or hybrid status (see Additional file 1: Figs. 1.7, 1.8 for genus-level comparisons where location-specific effects are not evident and all hybrid populations have significantly lower *β*-diversity than progenitor populations).

#### γ-Diversity

The majority of pairwise comparisons between single hybrid populations and single progenitor populations indicate that gut and skin *γ*-diversity is significantly higher in hybrid populations (see Fig. 1C, F; see Additional file 1: Fig. 1.9 for additional metrics). The sole exception is *γ*-diversity of the skin microbiota of hybrid lizards at SNW, which is intermediate to the two progenitor populations. Again, in this case, *γ*-diversity of the skin microbiota of both populations at SBluG is significantly higher than *γ*-diversity of the skin microbiota of both populations at SNW. Thus, similar to *β*-diversity, factors at the SBluG location appear to inflate *γ*-diversity of skin microbiota, potentially obscuring differences in *γ*-diversity due to lizard species or hybrid status.

#### Core diversity

**Fig. 2:**
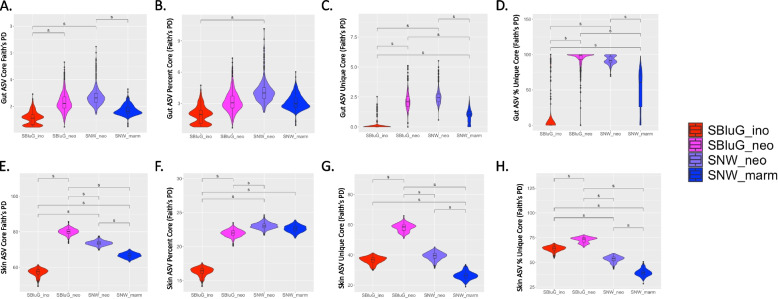
Diversity of the core gut (**A**, **B, C**, **D**) and skin (**E**, **F**, **G**, **H**) microbiota (ASVs) for *A. inornatus* from SBluG (red), *A. neomexicanus* from SBluG (magenta), *A. neomexicanus* from SNW (purple), and *A. marmoratus* from SNW (blue), assuming a 50% core threshold (i.e., to be considered part of the core, a microbial taxon must be present on at least half of the animals in a population). **A** and **E** show each population's core diversity. **B** and **F** show each population's percent core diversity. **C** and **G** show each population's core unique diversity. **D** and **H** show each population's percent unique core diversity. All panels are based on 500 bootstraps of 15 lizards from each population using the methods outlined in Chao et al [93, 94]. Significant differences, as determined by overlap of 83.4% confidence intervals, are indicated with an “s.” Additional information on the core microbiota, including analyses of alternative metrics and Venn diagrams, can be found in Additional file 2

Using a core threshold of 50% (i.e., to be part of the core, a microbial taxon must be present on at least half of the lizards in a population), the majority of pairwise comparisons between single hybrid populations and single progenitor populations indicate that core microbiota diversity is significantly higher in hybrid populations (see Fig. 2A, E; see Additional file 2: Figs. 2.1, 2.2, 2.3, 2.4). The sole exception is core diversity of the gut microbiota of hybrid lizards at SBluG, which is not significantly different from core diversity of the gut microbiota of progenitor lizards at SNW. Even in this case, however, median core diversity of the hybrid is still higher than median core diversity of the progenitor. Most pairwise comparisons between single hybrid populations and single progenitor populations do not indicate significant differences in the percent core diversity (i.e., percentage of *γ*-diversity that is present in the core) of the gut and skin microbiota. The exceptions are the percent core diversity of gut microbiota of hybrid lizards at SNW, which is significantly higher than the percent core diversity of progenitor lizards at SBluG, and the percent core diversity of skin microbiota of both hybrid populations, which is significantly higher than the percent core diversity of the progenitor population at SBluG. Notably, despite a lack of statistical significance, the median percent core diversity of the gut and skin microbiota is higher in hybrids than in progenitors for all but one comparison (skin microbiota of hybrid lizards at SBluG versus progenitor lizards at SNW).

#### Unique core diversity

At a core threshold of 50%, the majority of pairwise comparisons between single hybrid populations and single progenitor populations indicate that the unique core microbiota diversity is higher in hybrid populations (see Fig. 2C, G; see Additional file 2: Figs. 2.1, 2.2, 2.3, 2.4 for additional metrics). The sole exception is the unique core diversity of skin microbiota of hybrid lizards at SNW, which is not significantly different from the unique core diversity of skin microbiota of progenitor lizards at SBluG. Even in this case, however, the median unique core diversity of skin microbiota in hybrids is still higher than that of both progenitors. The majority of comparisons between single hybrid populations and single progenitor populations also indicate that the percent unique core diversity is higher in hybrid populations (see Fig. 2D, H; see Additional file 2: Figs. 2.1, 2.2, 2.3, 2.4 for additional metrics). The sole exception is the skin microbiota of the hybrid population at SNW, which is intermediate to the two progenitor populations. Similar to *β*- and *γ*-diversity, in this case, both SBluG populations are significantly higher than both SNW populations. Thus, factors at the SBluG location appear to inflate percent unique core diversity of the skin microbiota, potentially obscuring differences in the percent unique core diversity due to lizard species or hybrid status.

### Microbiota composition

We examine microbiota composition using two ordination space techniques. First, we consider the position of the hybrid centroid in ordination space based on maximizing system-wide variation using a UniFrac distance (principal coordinate analysis, PCoA). Second, we use the method in Mérot et al. [16] to consider position of the hybrid along and perpendicular to the main axis of variation between progenitors (“triangle plots”) [16]. For each technique, we separately consider hybrid outcomes (transgressive, conserved, intermediate) based on the position of the hybrid along each ordination axis (i.e., each PCoA axis or the axes parallel and perpendicular to the main axis of variation between progenitors).

#### PCoA

**Fig. 3:**
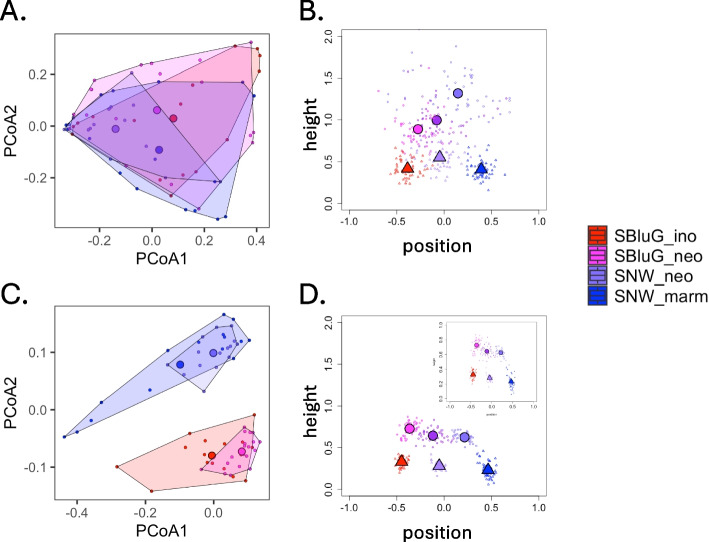
Two-dimensional PCoA (unweighted UniFrac distances) plots for the gut (**A**) and the skin (**C**) ASVs of *A. neomexicanus* from SBluG (magenta), *A. neomexicanus* from SNW (blue-purple), *A. marmoratus* (blue), and *A. inornatus* (red). Triangle plots of the hybrid mean microbiota for the gut (**B**) and skin (**D**) ASVs based on unweighted UniFrac distances. The position of the hybrid microbiota projection (*x*-axis) represents a measure of the distance between the hybrid and each progenitor along the main axis of parental variation, while the height of the triangle (*y*-axis) represents the deviation of the mean hybrid microbiota relative to main axis of parental variation. All values are calculated in the “microbiota space” defined by the first 20 principal coordinates (> 95% of variance) and normalized by the distance between progenitors. Large circles are the mean value of 50 subsamples on observed data, and small circles represent the outcome of each individual subsample on observed data. Large triangles are the mean value of 50 subsamples on null models, and small triangles represent the outcome of each individual subsample on a null model. For observed data, we considered the *A. neomexicanus* populations from both locations (purple), only the *A. neomexicanus* location from SNW (blue-purple), and only the *A. neomexicanus* location from SBluG (magenta). For null models, we consider subsamples of the *A. inornatus* population (red), subsamples of the *A. marmoratus* population (blue), and samples based on combining *A. inornatus* + *A. marmoratus* individuals (periwinkle). For both observed data and null models, subsamples consisted of 12 randomly sampled individuals from each progenitor population (see the “Materials and methods” section). Additional triangle plots can be found in Additional file 5; additional ordination plots can be found in Additional file 3

Pairwise comparisons between single hybrid populations and single progenitor populations show varied outcomes, depending on the PCoA axis and body site considered. Most pairwise comparisons between hybrids and progenitors do not indicate significant differences in gut microbiota along either the first or second PCoA axis (see Fig. 3A; see Additional file 3: Tables 3.3, 3.4). The sole exceptions are the hybrid population at SNW, which is significantly different from the progenitor population at SBluG along the first PCoA axis, and the hybrid population at SBluG, which is significantly different from the progenitor population at SNW along the second PCoA axis. Further, while the mean values of the gut microbiota of both hybrid populations are less than the mean values of the gut microbiota of both progenitor populations along the first PCoA axis, the differences are small. Results for skin microbiota are more complicated (see Fig. 3C; see Additional file 3: 3.11, 3.12 for additional metrics). About 50% of pairwise comparisons between single hybrid populations and single progenitor populations indicate significant differences. For example, the hybrid population at SBluG lies significantly higher along the first PCoA axis than either progenitor population. However, the hybrid population at SNW is not significantly different from either progenitor population along this same axis, though it does exhibit higher mean values. Along the second PCoA axis, neither hybrid population is significantly different from its syntopic progenitor, though both hybrid populations are significantly different from their the non-syntopic progenitors, and both hybrid populations have higher mean values than their syntopic progenitors (see Additional file 3 for more ordination analyses and Additional file 4 for PERMANOVA results that are broadly consistent with our PCoA results).

#### Triangle plots

Based on a 20-dimensional UniFrac projection, most pairwise comparisons between single hybrid populations and single progenitor populations indicate that hybrid microbiota are significantly different from and intermediate to progenitor null models (see “Materials and methods” section for a description of the progenitor null models) along the main axis of parental variation for both gut and skin microbiota (see Fig. 3B, D). The sole exception is gut microbiota of hybrid lizards at SNW, which is not significantly different from progenitor lizard null models at SNW. Even in this case, however, the mean value of the hybrid population at SNW lies intermediate to the mean values of the two progenitor null models. All pairwise comparisons indicate that hybrid populations are significantly different from progenitor populations along the perpendicular axis (i.e., the “height of the triangle”). Notice that because of the way the height of the triangle is defined (i.e., distance from a line drawn between the parental centroids), the height of the triangle will either be conserved (not different from progenitors) or transgressive (different from progenitors). It can never be intermediate (see Additional file 5 for additional metrics, 2D projections, and the microbial taxa that comprise the two axes of the triangle plots in Euclidean space).

### Microbial abundances

We consider microbial abundances of taxa that explain the most variation in our system, as identified based on a principal component analysis (PCA; see Additional file 3: Figs. 3.1 and 3.3 and Tables 3.1, 3.2, 3.9, and 3.10). Specifically, we select the microbial taxa with the highest loadings on the first two principal component axes for gut and skin microbiota. We then compare the relative abundances of these two microbial taxa in hybrids versus progenitor populations.

#### Dietzia maris

An ASV mapping to *D. maris* is the dominant loading (97.5%) on PC1 (49.98% of variation) for gut microbiota and is the dominant loading (97.33%) on PC2 (8.78% of variation) for skin microbiota. All pairwise comparisons between single hybrid populations and single progenitor populations indicate significantly lower abundance of the ASV mapping to *D. maris* in hybrid populations. Indeed, for both gut and skin microbiota, the median relative abundance of *D. maris* on hybrid lizards is zero, while it is non-zero on both progenitor populations.

#### Corynebacterium testudinoris

An ASV mapping to *C. testudinoris* is the dominant loading (94.27%) on PC2 (17.53% of variation) for gut microbiota. All pairwise comparisons between single hybrid populations and single progenitor populations indicate significantly lower abundance of the ASV mapping to *C. testudinoris* in the guts of hybrid animals. Similar to *D. maris*, the median relative abundance of *C. testudinoris* in gut microbiota of both hybrid populations is zero, while the median relative abundance of *C. testudinoris* in gut microbiota of both progenitor species is non-zero.

#### Fodinibacter luteus

**Fig. 4:**
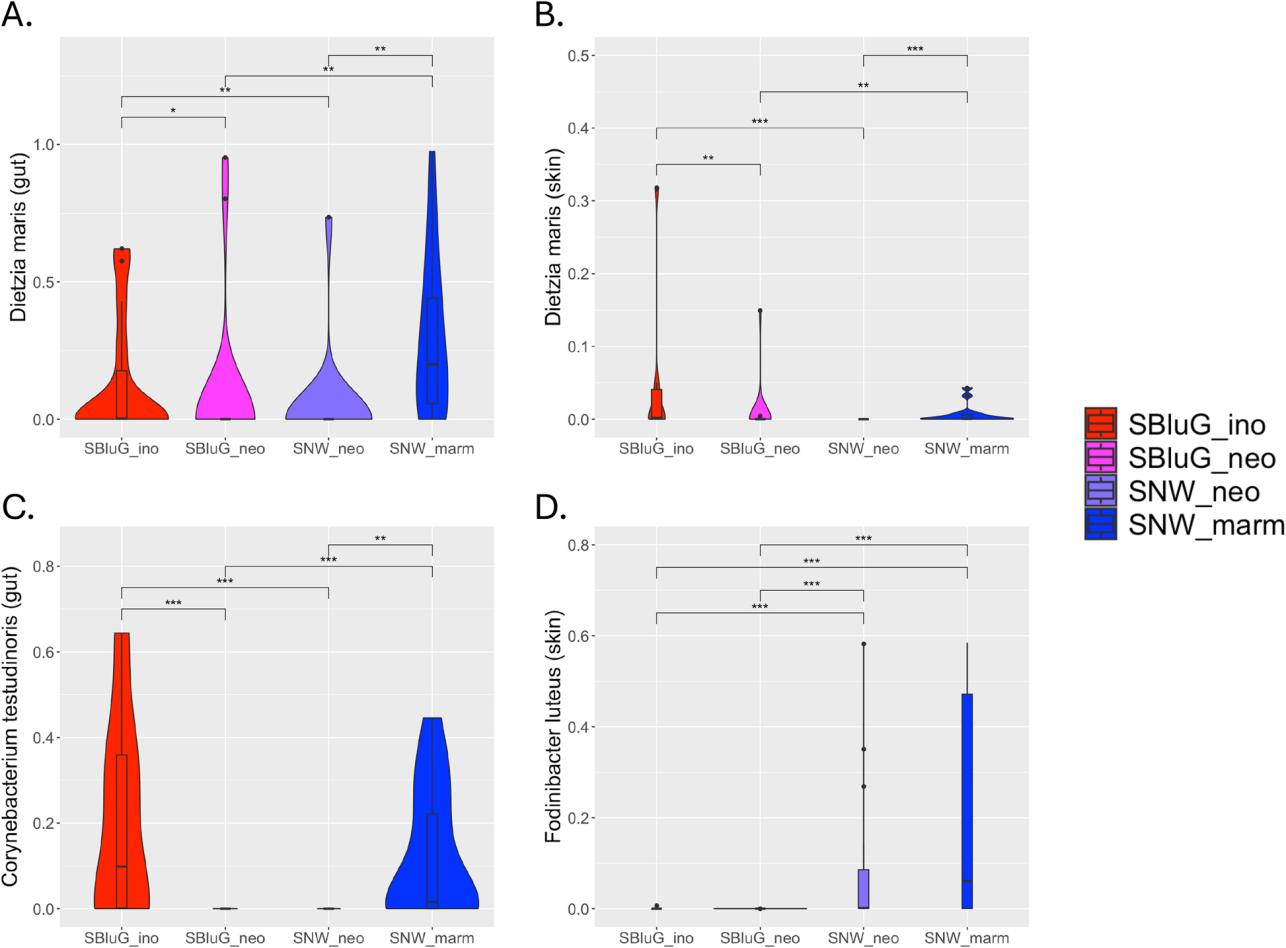
Relative abundances of ASVs representing the dominant loadings on gut and skin principal component axes (PCA). Taxa considered are as follows: *D. maris* in gut (**A**, dominant loading on PC1) and skin (**B**, dominant loading on PC2) microbiota, *C. testudinoris* in gut microbiota (**C**, dominant loading on PC2), and *F. luteus* in skin microbiota (**D**, dominant loading on PC1) for *A. inornatus* from SBluG (red), *A. neomexicanus* from SBluG (magenta), *A. neomexicanus* from SNW (purple), and *A. marmoratus* from SNW (blue)

An ASV mapping to *F. luteus* is the dominant loading (99.15%) on PC1 (73.99% of variation) for skin microbiota. The median relative abundance of *F. luteus* is higher in the skin microbiota of both lizard populations from SNW than it is in the skin microbiota of both lizard populations from SBluG (see Fig. 4D). However, there are no statistically significant differences in the relative abundances of *F. luteus* between either hybrid population and its syntopic progenitor. This suggests that location effects, rather than lizard species effects, explain why *F. luteus* is responsible for variation in our system.

Table 2 summarizes results for microbiota diversity, microbiota composition, and microbial abundance traits for each hybrid population. Specifically, we show outcomes (transgressive, conserved, intermediate) for each hybrid population compared to both progenitor populations and outcomes (differentiated/conserved) for each hybrid population compared to its syntopic progenitor population. We also show whether the outcomes are the same for both hybrid populations and whether trends in trait directionality (i.e., median/mean values of the trait independent of significance) are the same for both hybrid populations. Notably, for almost all traits, both hybrid populations differentiate from their syntopic progenitor and do so in the same direction. This is important because it suggests that our counts of transgressive segregation are likely conservative. In particular, in scenarios where one hybrid population differentiates significantly from both progenitors while the other hybrid population trends in the same direction but does not differentiate significantly from its non-syntopic progenitor, it is likely that the second population is, in fact, transgressive, but that location-specific effects are driving “apparent” trait conservation. For several other traits, transgressive segregation may be undercounted due to weak effect size and lack of statistical power. More specifically, in scenarios where the median/mean trait value of both hybrid populations trends in the same direction relative to progenitors, it is possible that the traits are actually weakly transgressive, but that detection of significance would require a larger sample size.

### Microbiota restructuring

Summarizing the HA microbiota with one- and two-dimensional metrics (e.g., diversity, microbial abundance, position in ordination space) is convenient for testing our hypotheses that hybrid HA microbiota traits should be distinct from and transgressive to progenitor HA microbiota traits. In reality, however, HA microbiota are highly multidimensional and thus are often difficult to fully summarize with simple one- and two-dimensional metrics. As a final analysis, we consider some of the more nuanced aspects of microbiota restructuring in the hybrid. This more holistic approach does not permit the simple hypothesis testing that we applied to the traits previously described but provides a summary of some of the subtler relationships between hybrid microbiota and the microbiota of progenitor species.

#### Microbial composition

**Fig. 5:**
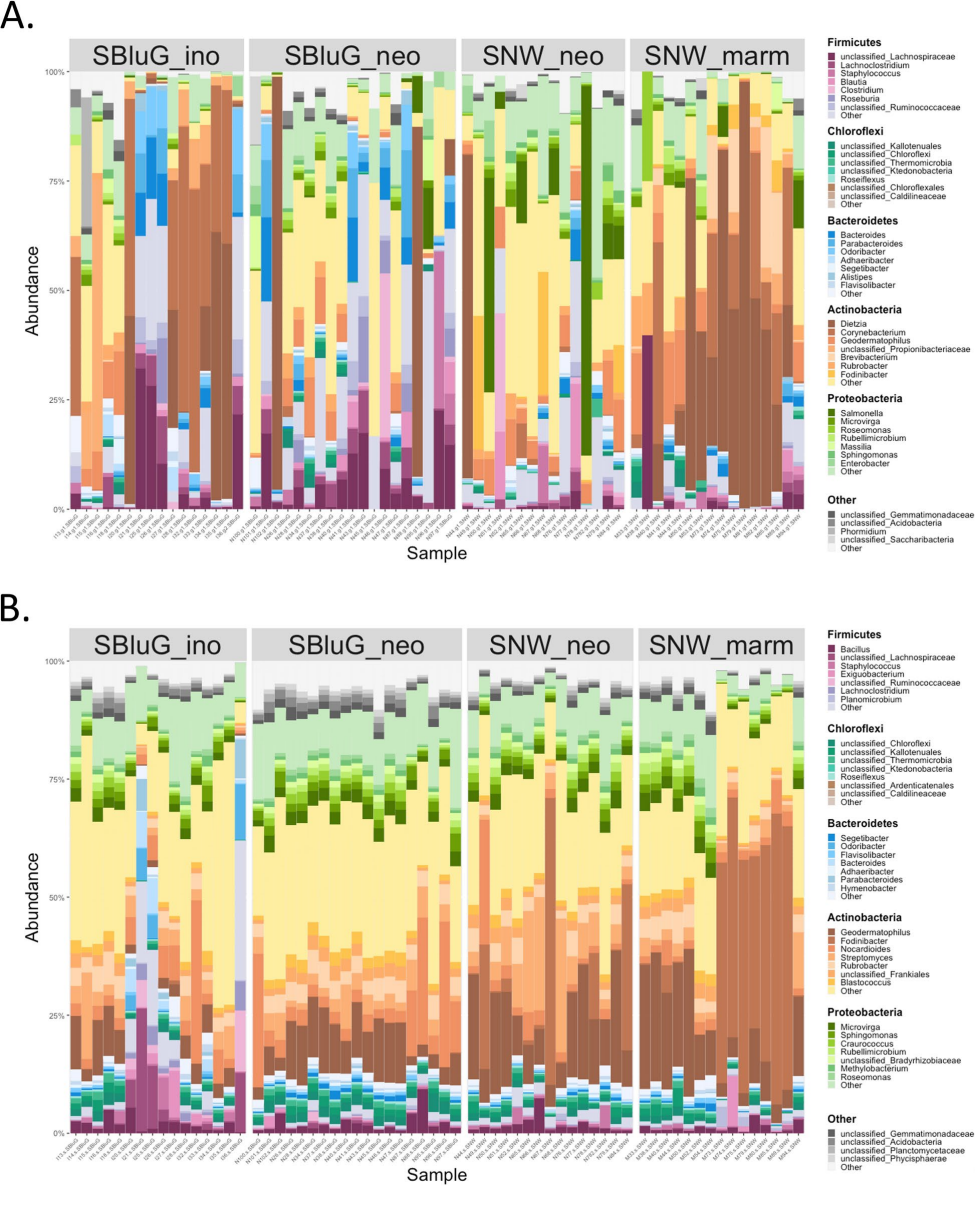
Bar graphs showing the genera composition of the gut (**A**) and skin (**B**) microbiota of each individual lizard in each population. See Additional files 6 and 7 for a description of dominant taxa and indicator species analysis

Figure 5 shows a summary of microbial taxa found on individual lizards. In Additional files 6 and 7, we also present a description of the general microbiota of each lizard species and identify taxa that are statistically over- and/or under-represented in hybrids relative to their parent species.

#### 4H index

**Fig. 6:**
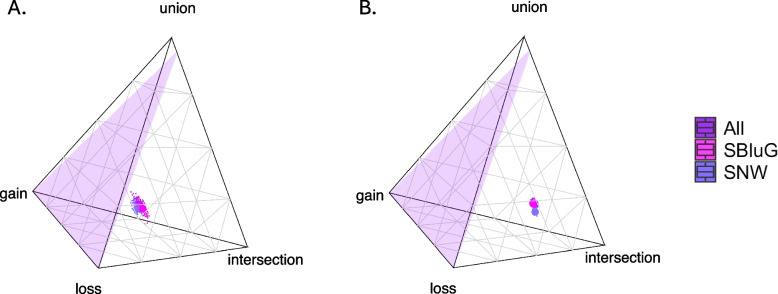
Quaternary plots of the 4H index for branch segments of the gut (**A**) and skin (**B**) microbiota of *Aspidoscelis neomexicanus* at SBluG (magenta), *A. neomexicanus* at SNW (blue-purple), and all *A. neomexicanus* at both locations (purple). The null plane is shown in purple in each plot. For 4H index values, see tables in Additional file 8

The 4H index [95] is a metric that we developed to describe restructuring of the hybrid microbiota according to four different models for how microbial taxa can be shared among progenitors and their hybrid offspring. Figure 6 shows a phylogenetic version of our 4H index applied to the full microbiota (i.e., a core threshold of 0). This allows us to quantify the extent to which hybrid microbiota (i) share microbial phylogenetic branches with the microbiota of both progenitors (intersection model), (ii) share microbial phylogenetic branches with the microbiota of one but not both progenitors (union model), (iii) are missing microbial phylogenetic branches found in the microbiota of one or both progenitors (loss model), and (iv) contain microbial phylogenetic branches not found in the microbiota of either progenitor (gain model). As compared to skin microbiota, gut microbiota of hybrid lizards include a higher proportion (see Additional file 8: Table 9.2) of microbial phylogenetic branches found on neither progenitor (gain model) and are missing a higher proportion of microbial phylogenetic branches found on one or both progenitors (loss model). By contrast, skin microbiota include a higher proportion of microbial phylogenetic branches found on both progenitors (intersection model). This results in gut microbiota showing approximately equal support for the “transgressive axis” (54%) and the “parental axis” (46%), whereas skin microbiota show much higher support for the “parental axis” (67%) relative to the “transgressive axis” (33%). Interestingly, hybrid animals are more likely than expected by chance to retain gut and skin microbial phylogenetic branches shared by both progenitors as opposed to microbial phylogenetic branches present on only one progenitor (see Additional file 8 for analysis of other metrics and core thresholds).

## Discussion

In this study, we explore the effects of hybridization on the HA microbiota in a parthenogenetic vertebrate (Table 1). Compared to previous studies, [31] our work has several advantages. First, and foremost, it considers HA microbiota in an ecologically successful hybrid. Further, it does so in a wild population, allowing microbiota to reflect both host genetics and, potentially, host environmental associations. As such, our study presents a full, in situ depiction of the hybrid microbiota. Finally, by using a vertebrate parthenogen, macromutations generated during F_1_ hybridization—arguably the most dramatic stage of the hybridization continuum (i.e., F_1_ hybridization → adaptive introgression → lineage reticulation)—are preserved. This makes hybrid parthenogens ideal natural laboratories for exploring the emergence of biological novelty [96] during hybridization and how this impacts hybrid ecology and fitness. In essence, hybrid parthenogens are clones of “hopeful monsters.” As such, they are useful simplified systems in which to examine how macromutations induced by F_1_ hybridization unlock novel ecological space (i.e., new adaptive zones [97]), which is a precursor to adaptive introgression.

### Hybrid trait outcomes

Across both gut and skin microbiota (see Table 1), we find more evidence for hybrid trait differentiation than for hybrid trait conservation (Hypothesis 1) and more evidence for transgressive traits than for intermediate traits (Hypothesis 2). This is striking because our estimates of transgressive segregation are conservative. Indeed, the true prevalence of transgressive traits is probably even higher than what we observe because some amount of transgressive segregation is likely obscured by location-specific effects and/or a lack of statistical power. Overall, our finding of widespread transgressive segregation in the microbiota traits of an ecologically successful hybrid organism suggests that transgressive holobiont traits are not restricted to dead-end hybrids but pervasive in successful hybrids as well.

### Microbiota diversity

Among diversity measures, *α*-diversity shows the weakest support for transgressive segregation (see Fig. 1A, D; Table 2). This is likely a result of weak statistical power and small effect sizes, since pooling hybrid populations into a single hybrid class and progenitor populations into a single progenitor class indicates significantly higher *α*-diversity of both skin and gut microbiota of hybrid animals (see Additional file 1: Figs. 1.4 and 1.6). Although our study is not designed to determine why hybrid microbiota have higher *α*-diversity, our results are consistent with previous findings that have correlated high allelic heterozygosity, like that observed in *Aspidoscelis* spp., [89] to high microbiota diversity [98, 99]. The higher *α*-diversity of hybrid microbiota is interesting because it could offer alternative explanations for hybrid vigor [100]. Typically, hybrid vigor is explained as the result of novel allelic combinations produced in newly heterozygous genomes (e.g., in hybrid progeny). Known as the heterosis hypothesis, the assumption is that these novel allelic combinations drive superior trait values such as faster growth rates or greater environmental tolerance through either dominance or overdominance mechanisms [101–103]. With respect to holobionts, hybrid vigor could emerge not only through dominance or overdominance in the host genome but also through dominance or overdominance in the broader hologenome [23]. There may, for example, be fitness benefits to inheriting two different microbial metagenomes because functions missing in one metagenome are present in the other (like masking of recessive alleles). Alternatively, there may be benefits to having microbial taxa that function in slightly different ways to perform similar tasks (complementation). Importantly, these “holobiont heterosis” (or “hybrid holobiont vigor”) mechanisms would require higher *α*-diversity in the hybrid microbial metagenome, which is what we observe.

The higher microbial *α*-diversity on individual hybrid lizards is also interesting in light of the general-purpose genotype (GPG) hypothesis. Formulated to explain the paradoxical success of asexual lineages, the GPG hypothesis postulates that successful clones persist because they exhibit greater ecological niche breadth, a more generalist ecological strategy, and/or greater phenotypic flexibility across one or more traits [104, 105]. Typically, this greater flexibility is attributed to differences in epigenetic regulation. [106, 107] Our results, however, suggest an alternative “general purpose metagenome” (GPMG) hypothesis. In particular, if functional diversity parallels taxonomic/phylogenetic diversity (an admittedly tenuous assumption), then the larger and more diverse microbial metagenomes of hybrid lizards may be at least partially responsible for conferring a more generalist ecological strategy and/or greater phenotypic flexibility to hybrid lizards.

As compared to *α*-diversity, *β*-diversity shows somewhat stronger support for transgressive segregation (see Fig. 1B, E; Table 2). However, results are still complicated by small effect sizes and, at least for skin microbiota, location-specific effects (although see transgressive genus-level comparisons unhindered by these factors in Additional file 1: Figs. 1.7, 1.8). Observing small effect sizes for *β*-diversity is not surprising. Consistent with many other HA microbial systems, [108–110] microbiota variation is extremely high within all lizard populations, with high turnover between individual lizards. As a result, *β*-diversity values of all four lizard populations are close to 1 (Fig. 1B, E), which limits differentiation between populations. Nevertheless, the fact that we see significant differences and the fact that all hybrid populations trend lower than progenitor populations, even when differences are not significant, suggest that hybrid animals likely exhibit less microbiota variation as compared to their progenitors. Several potential mechanisms could underlie the lower *β*-diversity of hybrids relative to their progenitors. First, in keeping with Lerner’s theory, [111] more diverse alleles of more heterozygous individuals might buffer against environmental perturbations, allowing for higher developmental stability. If Lerner’s theory applies to microbial phenotype (e.g., through a more diverse set of host MHC genes that buffer host–microbe interactions), then hybrid lizards should have more “stable” microbiota as compared to progenitors. Second, in keeping with the diversity-stability hypothesis, [112–115] the higher *α*-diversity of hybrid microbiota (presumably driven by higher host heterozygosity) may drive heightened microbiota stability (lower microbiota *β*-diversity). Though similar in its underlying driver (i.e., host heterozygosity), the diversity-stability hypothesis differs from the Lerner’s theory in that stability is not brought about directly by host–microbe interactions but rather by microbe-microbe interactions [101] (notice that both Lerner’s theory and the diversity-stability hypothesis require that we make a “space for time” substitution and assume that interindividual variation mimics temporal variation in a single individual). A final explanation is that the hybrid lizards are clones and thus exhibit relatively low interindividual genetic variation. In this case, it is not the high intraindividual host genetic diversity (i.e., high heterozygosity) but rather the low interindividual host genetic diversity that limits microbial variation among hybrids [116]. Without further study, it is impossible to confidently rule out any of the above three hypotheses, and the true cause could be a combination of these mechanisms or alternative mechanisms altogether.

*γ*-Diversity provides relatively strong support for transgressive segregation in hybrids, although results are again complicated by location-specific effects for skin microbiota (see Fig. 1C, F; Table 2). This is interesting because it indicates that the excess diversity conferred to progenitors by interindividual variation (i.e., *β*-diversity) is outweighed by the excess per individual diversity (i.e., *α*-diversity) of hybrids. Like *α*-diversity, the greater *γ*-diversity of hybrid populations has interesting implications for hybrid fitness and ecological success. In particular, it suggests that hybrid diversity (and putatively microbial functional diversity) is transgressive not only at the scale of individual animals (i.e., see GPMG hypothesis above) but also at the scale of entire populations.

Most measures of core diversity show strong support for transgressive segregation (see Fig. 2; Table 2), with hybrid populations having higher core diversity, higher unique core diversity, and higher percent unique core diversity. The transgressive nature of the hybrid core microbiota has several interesting implications. In particular, the higher unique core diversity and the higher percent unique core diversity in hybrids are of interest because they suggest that hybrids may have novel holobiont functionality [117, 118] not readily, or at least routinely, observed in their progenitors. Further, they suggest that hybrids may be more distinct from progenitors than progenitors are distinct from each other.

### Microbiota composition and microbial abundances

With respect to microbiota composition, we observe strong support for conservation of gut microbiota and mixed support for all three outcomes in skin microbiota (see Fig. 3; Table 2). The one compositional analysis where we do see overwhelming evidence of transgressive segregation, both in the hybrid gut and skin microbiota, is along the perpendicular axis of our triangle plots. By definition, this axis captures variation that does not differentiate progenitors; thus, it is arguably more likely to detect variation that separates hybrids from both progenitors. Despite the relatively low support for transgressive segregation in our compositional analyses overall, abundances of individual microbial taxa responsible for system-wide variation are often strongly transgressive, especially in the gut microbiota (see Fig. 4; Table 2). This suggests that individually transgressive microbial abundances are being combined in ways that lead to an overall conserved or intermediate microbiota composition. This could occur when the abundances of some microbial taxa are transgressive and higher on hybrids, while the abundances of other microbial taxa are transgressive and lower on hybrids. In this case, ordination techniques could project hybrids onto intermediate locations, even though hybrids are independently transgressive along all original axes in the system.

The complexities that emerge when attempting to understand a highly multidimensional set of traits using one-dimensional metrics or two-dimensional ordination axes highlight the importance of supplementing hypothesis testing on individual traits with a more holistic approach that can better capture the multidimensional nature of microbiota restructuring (Fig. 5). This can be done with our newly developed 4H index [95]. 4H analysis of our lizard system suggests restructuring of both gut and skin microbiota but finds stark body site differences in how restructuring occurs (Fig. 6). In particular, we see considerably more support for the gain (i.e., hybrids acquire novel microbes not found on either progenitor) and loss (i.e., hybrids are missing microbes found on one or both progenitors) models in gut microbiota (see Fig. 6), whereas skin microbiota are overwhelming dominated by the intersection (i.e., hybrids retain microbes found on both progenitors) model. As a consequence, the restructuring of gut microbiota falls more along the “transgressive axis” (i.e., gain–loss), whereas the restructuring of the skin microbiota falls more along the “parental axis” (i.e., intersection–union, conserved or intermediate). The two models characterizing the transgressive axis (gain and loss) are the foundation for rapid evolutionary change. The gain model, in particular, resembles saltation [119] or the generation of phenotypic novelty (“hopeful monsters”). By contrast, the parental axis and the intersection model, in particular, imply a likeness of hybrid microbiota to progenitor microbiota. This may contribute to ecological success if certain constituents of the progenitors’ skin microbiota are strongly beneficial for survival or if the loss of particular microbes disrupts host-microbiota symbioses [120–122]. In this case, retaining progenitor traits may be a requirement for lineage persistence.

### Hybrid microbiota and the hybrid niche

Our findings on *A. neomexicanus* microbiota have a number of interesting implications for holobiont ecology and evolution. In particular, some of the correlations that we observe between the *A. neomexicanus* microbiota and the *A. neomexicanus* niche suggests that there may be a relationship between hybrid microbiota restructuring and hybrid niche restructuring (assuming that functional changes parallel taxonomic changes). Very broadly, there are two reasons why hybrid microbiota might covary with hybrid niche. First, genetic restructuring of the hybrid might directly impact the hybrid microbiota, resulting in altered microbial function that then enables an altered hybrid niche (microbial cause). Second, genetic restructuring of the hybrid might directly impact the hybrid niche, resulting in different exposures to environmental microbes which then alters community reassembly of hybrid microbiota (microbial effect). In reality, interactions between host niche and the HA microbiota are likely bidirectional, [123, 124] with each affecting aspects of the other. Our study cannot be used to differentiate the directionality of restructuring (i.e., microbial cause = host genetics → host microbiota → host niche in contrast to microbial effect = host genetics → host niche → host microbiota). However, because of what we know about the ecology of *A. neomexicanus* relative to *A. inornatus* and *A. marmoratus*, our study *can* be used to discuss correlations between the hybrid niche and hybrid microbiota, a first step in determining whether host niche-HA microbiota interactions are occurring. In the following, we relate *A. neomexicanus* niche expansion and niche shift to *A. neomexicanus* microbiota phenotypes.

In general, the higher *α*- and *γ*-diversity of hybrid microbiota suggest a broader or expanded hybrid niche. This is true whether we consider a microbial cause or a microbial effect and is generally consistent with what is known about *A. neomexicanus* ecology. In particular, *A. neomexicanus* is capable of colonizing a wider breadth of habitats than either progenitor (see Additional file 9) [79, 87]. Under the microbial cause scenario, it is the greater hybrid microbial diversity that facilitates access to a wider range of environments and/or food sources. By contrast, under the microbial effect scenario, it is the use of a wider range of environments and/or food sources that alters microbiota assembly through exposure to a more diverse set of microbes. While the positive correlation between hybrid microbiota *α*-/*γ*-diversity and hybrid niche breadth could imply either a microbial cause or a microbial effect, the greater hybrid core diversity preferentially supports a microbial cause. This is because high core diversity implies conservation of excess microbial taxa across entire hybrid populations—a finding that is less likely if excess microbial taxa are purely a result of more diverse environmental exposures (i.e., an expansion of hybrid niche breadth). Indeed, if the larger core was driven solely by a wider range of environmental exposures, it would suggest that all individual lizards sampled the entire range of expanded microhabitats regularly enough to maintain a conserved set of environmental microbes associated with these exposures.

Whereas the excess diversity of the hybrid microbiota suggests niche expansion, the uniqueness of the hybrid microbiota suggests a niche shift. Notably, we see the uniqueness of the hybrid microbiota both in our core microbiota analysis and in the perpendicular axis of our triangle plots. PCoA analyses and PERMANOVA analyses (not shown, see Additional file 4: Tables 4.1, 4.2, 4.3, 4.4) also lend support to the distinctness of the hybrid microbiota, though not for all hybrid–progenitor comparisons. Again, a niche shift is consistent with what we know about *A. neomexicanus* ecology—that *A. neomexicanus* exhibits somewhat different dietary [125, 126] and microhabitat preferences [88] from its progenitors (even different macrohabitat preferences, see Additional file 9) [76, 79, 87]. As with diversity, the correlation between hybrid microbiota composition and the hybrid niche could reflect either a microbial cause or a microbial effect. Under the microbial cause scenario, it is the compositional restructuring of the hybrid microbiota that allows hybrids access to different resources, for example, prey items or microhabitats. By contrast, under the microbial effect scenario, it is altered environmental associations—and consequently, altered environmental exposures—which drive compositional restructuring of the hybrid microbiota. Unlike the niche expansion scenario, it is more difficult to speculate the extent to which microbial cause versus microbial effect drive the positive correlation between niche shift and a shift in microbiota composition.

### Implications for host-microbiota evolution

Whether ultimately due to a microbial cause or a microbial effect, correlations between the hybrid microbiota and hybrid niche indicate possible relationships between phenotypic novelty in the hybrid microbiota and ecological novelty that has previously been associated with hybrid ecological success. A broader host niche, for example, suggests a competitive advantage for the hybrid [127]. Meanwhile, a host niche shift indicates possible competitive release from progenitors [128, 129]. Observing potentially beneficial phenotypic novelty in the HA microbiota of our parthenogen model has important implications for how we should view the role of hybridization in holobiont evolution more broadly. Specifically, our findings suggest that transgressive HA microbiota traits (spanning single microbes to compositional phenotypes; see Tables 1 and 2) may function similarly to transgressive host traits with respect to their impacts on host evolution. Thus, transgressive microbiota traits produced during hybridization could impact host niches in ways that allow hybrid hosts to reach new adaptive zones [97]. This, in turn, could result in hybrids experiencing greater rates of evolution [130, 131] (potentially leading to adaptive radiations [132, 133]) or greater capacity for evolution (increased total diversification, morphological disparity, or ecological disparity [134–136]). If a transgressive microbiota trait enables occupation of a new adaptive zone and an increase in evolutionary capacity, then this trait, despite being of microbial origin, could be considered a host evolutionary/key innovation. [137] This would not be unprecedented. Indeed, some individual host–microbe symbioses have been previously identified as drivers of adaptive radiations [138, 139] and evolutionary innovations in hosts [140–142]. Still, the role of entire HA microbiota in adaptive radiations and key innovations of their host lineages has not been extensively discussed and should be explored as a potential mechanism for host evolution.

Beyond implications for host lineage evolution, hybrid microbiota restructuring could also impact evolution of HA microbes [143]. More specifically, host hybridization causes the reticulation of divergent microbial metagenomes. Admittedly, in the F_1_ generation, the resulting “hybrid microbial metagenomes” are not necessarily intermediate in genetic compositions (i.e., 50% paternal and 50% maternal) between the two progenitor microbial metagenomes from which they arose. Still, in most cases, they likely represent combinations of microbial metagenomic components from both progenitor species. Such microbial metagenome restructuring has the potential to alter microbiota function by introducing novel microbe–microbe interactions, novel host–microbe interactions, or even novel microbes entirely (e.g., the colonization of niches left unoccupied or newly created). Over evolutionary timescales, this microbial “metagenome hybridization” could contribute to reticulate evolution of the microbial metagenome. Interestingly, because the ratio of progenitor microbial metagenomes does not necessarily parallel the ratio of progenitor genomes in hybrid holobionts, reticulate evolution may occur asymmetrically between the host genome and microbial metagenome. Differences in patterns of gene flow between host genomes and their microbial metagenomes may have significant implications for how we understand holobiont/hologenome evolution, both within the context of host hybridization and more broadly.

Finally, host hybridization could even increase the capacity for evolution of individual microbial genomes. In particular, when microbiota are suddenly and dramatically reorganized into communities containing novel combinations of microbial taxa, the capacity for microbial novelty within existing microbial taxa, both as a result of altered selection forces imposed by ecological opportunity and via new opportunities for horizontal gene transfer, likely increases. This is consistent with previous applications of the hopeful monster concept [48] to the rapid evolution of antibiotic resistance due to horizontal gene transfer [144]. Importantly, over longer timescales, new ecological opportunities and horizontal gene transfer could lead to microbial adaptive radiations [145, 146], evolutionary/key innovations, [147, 148] and ultimately, reticulate evolution [149] (i.e., horizontal gene transfer) within the phylogenies of individual microbial taxa.

The simultaneous impact of host hybridization across multiple scales within the hologenome (i.e., host genome, microbial metagenome, and microbial genome) suggests extensive opportunity for phenotypic novelty, some of which may lead to fitness benefits or niche changes at their respective scale, across multiple scales, or even at the scale of the entire holobiont. Thus, the multiscale nature of holobiont hybridization gives rise to a much wider range of genetic combinations and genetic interactions than are possible with host genomes alone. Despite this, whether and how scales of hybridization interact to generate hopeful holobionts and their implications for eco-evolutionary success remain open questions.

### Caveats and future directions

Despite the many advantages of our hybrid parthenogen system, a range of extensions to our existing work could prove interesting. One challenge of our study was location-specific effects. Discovery of rare locations of triple syntopy, or broader sampling of hybrid and progenitor microbiota across the full spectrum of habitats where they co-occur, could help to address challenges where outcome interpretation was potentially impacted by location-specific effects. Further, some of the traits that we considered appeared to have small effect sizes, making it difficult to detect significant differences with 15 animals from each population. In future studies, collecting microbiome samples from a larger number of animals could help to resolve our understanding of hybridization on several holobiont traits in the *A. neomexicanus* system.

Few other hybrid microbiota studies in ecologically successful systems have characterized the HA microbiota in situ [41]. While that is an advantage of our study, future work should house hybrid and progenitor species under identical conditions in a captive setting to separate microbial causes from microbial effects as the underlying drivers of microbiota differences between species. Another caveat in our study is that we focus on broad-scale differences in microbiota taxonomy. Without knowing the functional consequences, we are unable to directly relate the hybrid holobiont traits that we observe to potential ecological impacts on the host. Now that we have demonstrated significant and transgressive changes in the hybrid HA microbiota, including changes that broadly parallel the hybrid niche, it would be interesting to address potential functional changes. Therefore, shotgun metagenomic sequencing or even culture experiments on some of the dominant strains that we observe driving variation across species (e.g., *D. maris*; see Additional files 6 and 7) are important future directions.

Another complication of our study is that we assume *A. neomexicanus* represents a frozen F_1_ hybrid, and that we are, thus, isolating the front-end effects of hybridization. However, *A. neomexicanus* originated in the Pleistocene, allowing time for the accumulation of genetic mutations [89]. This could result in the sampled *A. neomexicanus* population exhibiting differentiation from the true F_1_ offspring of the original *A. marmoratus* × *A. inornatus* cross. While the persistence of *A. neomexicanus* since the Pleistocene reflects its ecological success, we cannot rule out the possibility that additional evolutionary processes since its hybrid origin have contributed to the microbiota differences that we observe between *A. neomexicanus* and its progenitors. Further, because parthenogenetic *Aspidoscelis* evolution is constrained by their clonal reproductive mode, these systems are not ideal for investigating every question related to the hybridization continuum and how it connects microevolutionary process to macroevolutionary pattern. For example, hybrid parthenogens, as front-end systems, have limited utility for understanding the role of hybridization at deeper evolutionary scales such as ongoing adaptive introgression [150, 151] and reticulate phylogenetics [149]. “Back-end” hybrid systems (i.e., those exhibiting considerable backcrossing) by contrast, including sexually reproducing hybrid *Aspidoscelis* systems, [89, 152] are much better models for investigating these scales. Indeed, pairing successful front-end and back-end systems will ultimately be key for unraveling hybridization’s role as a mode of holobiont evolution.

## Conclusion

We found considerable evidence of transgressive phenotypes in both the gut and skin microbiota of the ecologically successful hybrid, *A. neomexicanus* (see Tables 1 and 2). Our findings raise important questions about the nature of host–microbiota interactions in hybrid animals and their relevance to the ecological and evolutionary success of the holobiont. Exploring the bidirectional effects of hybridization on host niche and the HA microbiota, as well as teasing apart the microbiota reassembly mechanisms responsible for these relationships, mark exciting future prospects for hybrid microbiota research. Goldschmidt’s lasting legacy was his realization that hopeful monsters have disproportionately positive impacts on evolutionary trajectories. Since Goldschmidt, hybridization has become a widely accepted driver of macrobial adaptive radiation, macrobial evolutionary innovation, and evolutionary diversification more broadly. Holobiont research, however, has largely neglected hybridization as an important driver of evolutionary success. This is because existing studies have found primarily negative consequences for host–microbe and microbe–microbe interactions. [31, 39, 40] Aligning holobiont research with broader evolutionary paradigms requires understanding that the numerous hybrid holobiont systems with mismatched microbiota, host–microbe incompatibilities, and/or immune system dysregulation do not negate the importance of hybridization to positive evolutionary outcomes. Rather, host hybridization can still be important because it is the rare instances of ecological success—the hopeful holobionts—which have outsized benefits on holobiont evolution.

## Materials and methods

### Species description, location description, and animal capture

We used a hybrid lizard (*Aspidoscelis* spp., Teiidae) system broadly distributed throughout Central New Mexico, USA. More specifically, we sampled adults representing the hybrid, obligately parthenogenetic species *A. neomexicanus* where it occurred syntopically with its two progenitor species: *A. inornatus* (♂, also considered *Aspidoscelis arizonae* [69, 89]) and *A. marmoratus* (♀) [68]. We chose *A. neomexicanus* as our focal hybrid system because it is broadly distributed across diverse habitats, is known to occur syntopically with both progenitor species, [79, 87, 88] is of recent hybrid origin, and, to the best of our knowledge, is representative of a single hybrid origin [89]. Note that we follow taxonomic recommendations by Tucker [153] and Walker et al. [71] and use the masculine species epithet for *Aspidoscelis* spp.

All *Aspidoscelis* lizards were captured from two locations within Sevilleta National Wildlife Refuge in Socorro County, NM, USA between late May and early August, 2022. *A. marmoratus* and *A. neomexicanus* occurred syntopically at the “Sevilleta Northwest” (referred herein as “SNW”) location (34.397882, − 106.867637, Decimal Degrees WGS84), while *A. inornatus* and *A. neomexicanus* occurred syntopically at the “Sevilleta Blue Grama” (referred herein as “SBluG”) location (34.331178, − 106.636717, Decimal Degrees WGS84). Lizards were captured either by hand or using drift fences with box funnel traps. All animal bycatch was immediately released from traps upon capture (see Camper et al. [154] for further location details [where locations SNW = S-NW and SBluG = S-NE] and notes on our trapping methodology). Locations were sampled until we caught at least 15 female lizards of each whiptail species.

### Animal sampling

Following animal capture, we collected a single skin swab and a single cloacal (“gut”) swab to sample the microbiota at each body site. For skin swabs, lizards were washed with sterile millipore water for ca. 30 s. Lizards were then swabbed (sterile Puritan HydraFlock no. 253406H; tip style large; tip dimensions 1.6 cm [0.64 in.] length × 0.55 cm [0.21 in] diameter) along each major surface 15–20 times (dorsal surface of the cranium, dorsum, laterally in between limbs, dorsal and ventral surfaces of all extremities including tail, venter, and throat) avoiding the mouth and cloaca. For cloacal swabs, the lizards’ cloaca were wiped with a 70% isopropyl ethanol pad for ca. 30 s, and a swab (sterile Puritan HydraFlock no. 253318H; tip style Micro Ultrafine; tip dimensions 0.7 cm [0.31 in] length × 0.3556 cm [0.14 in] diameter) was inserted into the cloaca and gently twisted and pulsed back-and-forth along the anterior–posterior axis for ca. 30 s more. Each swab was deposited in a separate 2-mL DNA/RNA shield tube (Zymo, no. R1102) immediately after collection. All lizards were handled using nitrile gloves wiped down with 70% isopropyl alcohol throughout the entire sampling process. We only used female lizards in our data analysis and confirmed their sex by attempting to evert the hemipenes using gentle pressure applied ventrolaterally ca. 0.5 cm posterior to the cloaca. Before releasing sampled lizards 50–100 m away from their capture location (if captured using traps), we marked each lizard with a unique identifier using a battery-powered miniature medical cautery unit (see Additional file 10: Fig. 10.1 regarding our marking scheme). This was done to prevent duplicate sampling of the same lizard.

### 16S rRNA gene sequencing

All samples were sent to ZymoBIOMICS Targeted Sequencing Service for Microbiome Analysis (Zymo Research) using 16S rRNA gene sequencing. Below we outline the basic methods that were used. Additional details are available from https://www.zymoresearch.com/pages/16s-its-amplicon-sequencing.

#### DNA extraction

Gut and skin microbiota samples were extracted using the ZymoBIOMICS®−96 MagBead DNA Kit (Zymo Research, Irvine, CA, USA) using an automated platform.

#### Targeted library preparation

The 16S ribosomal RNA gene was targeted using custom primers (*Quick*−16STM Primer Set) to amplify the V3–V4 region. The *Quick*−16S™ NGS Library Prep Kit was used for library preparation, and libraries were amplified using real-time PCR. Final PCR products were quantified with qPCR fluorescence readings and pooled based on equal molarity. PCR libraries were cleaned with the Select-a-Size DNA Clean & Concentrator™ and then quantified using TapeStation® (Agilent Technologies, Santa Clara, CA, USA) and Qubit® (Thermo Fisher Scientific, Waltham, WA, USA).

#### Control samples

A positive control (ZymoBIOMICS® Microbial Community Standard) was used for each DNA extraction and targeted library preparation. Blank or negative controls were used for each extraction and library preparation. In addition to these standard controls, in the field, we collected a series of environmental controls including air (*N* = 2), sterile millipore water (*N* = 1), soil (*N* = 2), box funnel traps (*N* = 2), and a Chevrolet Express 1500 dashboard (*N* = 1). Air samples were collected using the small swab (aforementioned sterile Puritan HydraFlock no. 253318H), and all other control samples were collected using the large swab (aforementioned sterile Puritan HydraFlock no. 253406H).

#### Sequencing

An Illumina® MiSeq™ was used to sequence the final library with a V3 reagent kit over 600 cycles. Sequencing was performed with a 10% PhiX spike-in.

#### Bioinformatics analysis

We used the metadata and biom files provided to us by the ZymoBIOMICS Targeted Sequencing Service (available on our GitHub page: https://github.com/bewicklab/HopefulHybridHolobionts/) for all subsequent analyses. Briefly, Zymo Research generates these files by inferring unique ASVs from raw reads, removing potential sequencing errors, and removing chimeric sequences using the DADA2 pipeline [155]. They then assign taxonomy to the dataset using Uclust (Qiime v1.9.1) with reference to the Zymo Research Database—a 16S database curated by Zymo Research. Finally, we used Qiime2 to generate a phylogenetic tree for all microbial ASVs based on the 16S rRNA sequences provided by Zymo Research. The sequence files and script for tree generation are available on our GitHub page. Control samples were not used in analysis, but associated FASTQ files have been made publicly available at the National Center for Biotechnology Information Sequence Read Archive (BioProject accession number PRJNA1188019; sample accession numbers SRR31414743–SRR31414887; https://www.ncbi.nlm.nih.gov/bioproject/1188019).

### Statistical analyses

For all analyses (both gut and skin), we applied statistical tests to both amplicon sequence variants (ASVs) and 16S rRNA gene sequences pooled to microbial genus. Running analyses at two different taxonomic scales allows for assessment of the effects of taxonomic scale. For ASV analyses, we considered both phylogenetically aware and phylogenetically agnostic methods. For genus-level analyses (both the gut and skin), we only considered phylogenetically agnostic methods. For both ASV and genus-level analyses (both gut and skin), we considered both incidence-based and abundance-based approaches. In the main text, we present results for analysis of ASVs using phylogenetically aware incidence-based measures. This is because abundance-based methods cannot be leveraged for metrics that calculate overall diversity across multiple animals (e.g., *γ*-diversity, the core microbiota). Further, the phylogenetically aware methods applied to ASVs represent an intermediate scenario that interpolates between focusing on strain-level variation versus focusing on large-scale phylogenetic differences among microbiota. Other analyses, however, are presented in the additional files because these provide different insights into the types of changes that emerge in the microbiota of hybrid animals.

For all analyses, we first removed 16S rRNA gene sequences that could not be identified as Bacteria or Archaea based on the proprietary Zymo database. These sequences were often associated with long branch lengths in the microbial phylogenetic tree and likely represented sequencing artifacts and non-microbial contamination. Additionally, we removed all samples with a read depth lower than 10,000 reads. This resulted in the loss of three gut samples but no skin samples, leaving 19 *A. neomexicanus* gut and skin samples at SBluG, 15 *A. neomexicanus* gut and skin samples at SNW, 16 *A. marmoratus* at SNW, 15 *A. marmoratus* skin samples at SNW, and 16 *A. inornatus* gut and skin samples at SBluG. Finally, we rarefied all samples to the read depth of the lowest sample from each particular body site (13,126 reads for gut samples, 116,931 reads for skin samples) using the rarefy_even_depth function from the phyloseq package [156]. All analyses were performed using the R programming language (version 4.2.1) [157], and associated code is available on our GitHub page (see the “Bioinformatics analysis”).

#### α-Diversity

For gut and skin ASVs, we considered richness (i.e., a count of ASVs), Shannon diversity, Simpson diversity, Faith’s phylogenetic diversity (PD), and Rao’s quadratic entropy. For microbial genera, we considered richness, Shannon diversity, and Simpson diversity. Richness was calculated by counting microbial presences in the ASV and genus tables for each sample. Shannon and Simpson diversities were calculated using the diversity function in the vegan package (version 2.6.4) [158]. Faith’s PD was calculated using the pd function in the picante package (version 1.8.2) [159]. Rao’s quadratic entropy was calculated using the raoD function in the picante package. For Faith’s PD, we used a rooted, non-ultrametric microbial phylogeny. However, because the raoD function requires an ultrametric tree, for Rao’s quadratic entropy, we converted the microbial phylogeny into an ultrametric tree by first removing multichotomies using the multi2di function and then applying molecular dating with mean path lengths using the chonoMPL function, both from the ape package (version 5.7.1) [160]. To identify significant differences in *α*-diversity between groups, we first used a Kruskal–Wallis test applied to the four distinct populations (*A. inornatus* at SBluG, *A. neomexicanus* at SBluG, *A. neomexicanus* at SNW, and *A. marmoratus*at SNW). This was done using the kruskal.test function from the stats package (version 4.2.1) [157]. When the Kruskal–Wallis test was significant, we did pairwise post hoc testing by applying pairwise Wilcoxon rank-sum tests with a Benjamini–Hochberg correction using the pairwise.wilcox.test function, also from the stats package. In addition to testing for diversity differences between the four populations separately, we also tested for diversity differences between asexual (both hybrid populations) and bisexual (both progenitor populations) lizards. For asexual/bisexual comparisons, all statistical tests were performed as described above, except that we used a Mann–Whitney *U*-test in place of a Kruskal–Wallis test. This was done with the wilcox.test function from the stats package. All animals available from each population were used in analysis of *α*-diversity.

#### β-Diversity

For all estimates of *β*-diversity (gut and skin), we used multisite dissimilarity indices [161, 162]. We chose this approach over pairwise or averaged pairwise dissimilarity indices because multisite dissimilarity indices avoid pseudoreplication (a problem with pairwise metrics) and account for higher-order co-occurrences (a problem with averaged pairwise metrics). Thus, multisite dissimilarity indices better represent dissimilarity across larger (> 3) sets of communities [161]. For ASVs, we considered Jaccard and unweighted UniFrac dissimilarity indices. For microbial genera, we considered Jaccard dissimilarity index. To test for significant differences between groups, we used a bootstrap approach [93, 94, 163] to determine 83.4% confidence intervals (CIs). When CIs between any two populations did not overlap, we assumed that the populations were significantly different. We used overlap of 83.4% CIs because this corresponds to a *p*-value of approximately 0.05. [164] Briefly, for each of our four distinct populations (*A. inornatus* at SBluG, *A. neomexicanus* at SBluG, *A. neomexicanus* at SNW, and *A. marmoratus* at SNW), we generated a “bootstrap assemblage” based on the methods in Chao et al [93, 94, 163]. We then used this “bootstrap assemblage” to create 500 bootstraps of *N* = 15 lizards, and for each bootstrap, we calculated the multisite *β*-diversity. *β*-diversity was calculated using the beta.multi and the phylo.beta.multi functions from the betapart package (version 1.6) [165] for Jaccard and unweighted UniFrac dissimilarities respectively. To assess the significance of differences between populations, we sorted bootstrap values of *β*-diversity for each population and took the upper and lower CIs for the population as the 417th (83.4th percentile) and 83rd (16.6th percentile) bootstrap values of *β*-diversity respectively.

#### γ-Diversity

For *γ*-diversity, we again used a bootstrap approach for both gut and skin. Specifically, we created bootstrap assemblages for each lizard population, used these to generate 500 bootstraps of *N* = 15 lizards, and then calculated estimates of *γ*-diversity for each bootstrap (see *β*-diversity for details). For ASVs, we calculated overall *γ*-diversity based on richness (i.e., a count of all of the microbial taxa present across all 15 animals) and Faith’s PD. For microbial genera, we only considered richness. Similar to *α*-diversity, we used the pd function in the picante package to calculate Faith’s PD. As with *β*-diversity, we assessed significance of differences between populations based on overlap of 83.4% CIs.

#### Core diversity

Similar to *β*- and *γ*-diversity, we used a bootstrap approach to examine the properties of the gut and skin core microbiota for each lizard population. As before, we generated bootstrap assemblages and created 500 bootstraps of *N* = 15 lizards from each population. We then identified the core microbiota for each bootstrap of each lizard population. For this analysis, we used a 50% core threshold, meaning that a microbial taxon had to be present on 50% of hosts (eight hosts) in a population to be considered core. For ASVs, we calculated the richness (i.e., a count of all the microbial taxa present) and the Faith’s PD of the core microbiota, and for genera, we calculated the richness (i.e., a count of all the microbial genera present). We also determined what percentage of the overall *γ*-diversity was represented in the core (“percent core diversity”) by dividing core diversity by *γ*-diversity. Further, we calculated the ASV richness, Faith’s PD, and genus richness of the unique component of the core microbiota of each population (“unique core diversity”). For the two progenitor populations, “unique” was defined as any microbial taxon not found in the core microbiota of any other population (though potentially present but not part of the core in another population). For the hybrid populations, “unique” was defined as any microbial taxon not found in the core microbiota of either progenitor population, regardless of whether or not it was present in the core of the other hybrid population Finally, we calculated the percentage of the core microbiota that was unique to any given population (“percent unique core diversity”) by dividing the richness/diversity of the unique component of the core microbiota by the richness/diversity of the entire core microbiota of that population. The significance of differences between groups was again assessed using overlap of 83.4% CIs (see *β*-diversity section for details). For all analyses of unique core microbes, our bootstrap assemblages consisted of only observed taxa. Although an approximation, we found no undetected taxa in any of the 500 trials for core gut microbiota of each lizard population and found 2–3 trials with a single undetected taxon out of 500 trials for the core skin microbiota of each lizard population. Because we do not know the identity of these undetected taxa, it is impossible to determine whether or not they are unique. However, given their extremely low prevalence, ignoring these taxa should have a negligible effect on our results. Venn diagrams were similarly made by defining the core microbiota as any microbial taxon that was part of the microbiota of at least 50% of hosts of any given population. Venn diagrams were generated based on the entire dataset (i.e., not using resampling) using the venn.diagram function in the VennDiagram package (version 1.7.3) [166, 167].

#### Triangle plots and ordination

For both gut and skin, triangle plots and principal coordinate analysis (PCoA) were performed on Euclidean, Jaccard, Bray–Curtis, unweighted UniFrac, and weighted Unifrac distances for ASVs, as well as Euclidean, Jaccard, and Bray–Curtis distances for microbial genera. Triangle plots and associated null models were generated using the TriangleHbootstrap, and TriangleHnull functions from the HybridMicrobiomes package subsampling *N* = 12 lizards for each trial. Briefly, triangle plots are generated by locating the progenitor centroids and the centroid of the hybrid population(s), finding the projection of the hybrid centroid onto the axis connecting the parental centroids (i.e., “position along the main axis of parental variation”), and then finding the perpendicular distance between the parental axis and the hybrid centroid (i.e., “triangle height”). For abundance-based metrics (i.e., Euclidean, Bray–Curtis, and weighted UniFrac), we generated a hybrid null model by averaging microbial abundances from pairs of randomly selected *A. marmoratus* and *A. inornatus* individuals. For incidence-based metrics (i.e., Jaccard and unweighted UniFrac), we generated a hybrid null model by selecting a random pair of *A. marmoratus* and *A. inornatus*, recording the presence of microbial taxa on both progenitors and randomly recording half of the presences of microbial taxa found on either progenitor but not both. Progenitor null models were generated by randomly sampling individuals from the *A. inornatus* and *A. marmoratus* populations respectively. For more details, see function descriptions in the HybridMicrobiomes package [95]. PCoA was performed using the pcoa function from the ape package [160]. For triangle plots, differences between hybrid and progenitor microbiota were assessed using the TriangleHcompare function individually on the *x*-axis (position) and *y*-axis (height). For PCoA analyses, differences between hybrid and progenitor microbiota were assessed by applying Mann–Whitney *U*-tests to the hybrid and progenitor populations separately based on position along the first and second principal coordinate axes.

#### Microbial abundances

To compare individual microbial relative abundances across populations, we used principal component analysis (PCA) and selected the microbial taxa with the dominant loadings on the PC1 and PC2 axes for gut and skin respectively. For gut microbiota, the two taxa identified were ASVs corresponding to *D. maris* and *C. testudinoris*. For skin microbiota, the two taxa identified were ASVs corresponding to *D. maris* and *F. luteus*. To identify significant differences in the relative abundances of these microbial taxa between groups, we first used a Kruskal–Wallis test applied to the four distinct populations (*A. inornatus* at SBluG, *A. neomexicanus* at SBluG, *A. neomexicanus* at SNW, and *A. marmoratus* at SNW). This was done using the kruskal.test function from the stats package (version 4.2.1). [157] When the Kruskal–Wallis test was significant, we did pairwise post hoc testing by applying pairwise Wilcoxon rank-sum tests with a Benjamini–Hochberg correction using the pairwise.wilcox.test function, also from the stats package. All animals available from each population were used in analysis of microbial abundances.

#### Microbiota restructuring

To visualize microbiota restructuring for both gut and skin genera, we created a stacked bar graph (microshades package; [168] version 1.13) to observe the relative abundance of each genus. Further, for both gut and skin microbiota, quaternary plots were generated by defining the core microbiota as any microbial taxon or (for the UniFrac-inspired 4H metric) microbial phylogenetic branch segment that was part of the microbiota of a fixed number of hosts of any given population. For the main paper, we consider a core threshold of 0% (i.e., the full microbiota). However, we also present analysis using a 50% core threshold in additional file 8. For Jaccard-inspired 4H indices, we used the FourHbootstrap, FourHquaternary, and FourHnullplane functions from the HybridMicrobiomes package (version 0.1.2) [95]. Because the HybridMicrobiomes package does not include a UniFrac-inspired 4H index, we developed this for the current study. Briefly, using the phylogenetic tree for the entire system (i.e., the microbiota of both progenitors and the hybrid), a branch segment in the phylogenetic tree was determined to be part of the core of a particular population or species if it was present on at least the threshold number of animals of that population/species. Once core branch segments had been determined for all populations/species, the populations were compared. Intersection was taken as the fraction of the total branch length shared between the hybrid and both progenitors. Union was taken as the fraction of the total branch length shared between the hybrid and one but not both progenitors. Gain was taken as the fraction of the total branch length only found on the hybrid, and loss was taken as the fraction of the total branch length not found on the hybrid but found on at least one progenitor. This is analogous to our previous definition of the 4H index [95] but uses branch lengths instead of taxon counts. To fully characterize the range of possible outcomes for the 4H index, we used 100 subsamples of 12 animals from each population, with the hybrid populations either including only the animals from SBluG, only the animals from SNW, or both. In order to ensure comparability between gut and skin microbiota, we rarefied all samples to the lowest read depth present in our gut dataset.

## Supplementary Information

Additional file 1. Supplementary analyses for microbiota diversity used in this study including figures 1.1-1.9.

Additional file 2. Supplementary analyses for core microbiota diversity used in this study including figures 2.1-2.5.

Additional file 3. Supplementary analyses of ordination plots used in this study including figures 3.1-3.4 and tables 3.1-3.16.

Additional file 4. Supplementary analyses of PERMANOVA used in this study including tables 4.1-4.14.

Additional file 5. Supplementary analyses of triangle plots used in this study including figures 5.1-5.4 and tables 5.1-5.14.

Additional file 6. Supplementary analyses of dominant taxa used in this study including tables 6.1-6.4.

Additional file 7. Spreadsheet tabs providing output of indicator species analysis for microbial taxa distinguishing each species and hybrids vs. progenitors.

Additional file 8. Supplementary analyses of the 4H index used in this study including figures 8.1-8.2 and tables 8.1-8.4.

Additional file 9. Supplementary analyses of habitat niche in the *Aspidoscelis neomexicanus *complex used in this study including figures 9.1-9.2.

Additional file 10. Supplementary information for lizard marking method implemented in this study including figure 10.1.

Additional file 11: BIOM files, read counts, taxonomy assignments, microbial phylogenies, and lizard metadata for gut and skin samples used in this study.

## Data Availability

All sequence data has been deposited to the National Center for Biotechnology Information Sequence Read Archive, available at https://www.ncbi.nlm.nih.gov/bioproject/1188019 (BioProject accession number PRJNA1188019; sample accession numbers SRR31414743–SRR31414887). BIOM and metadata files are included in Additional file 10. Additionally, BIOM files, metadata files, and all code necessary for the analyses presented in this manuscript are available at https://github.com/bewicklab/HopefulHybridHolobionts/.
