# Supplementary material for: Transgressive hybrids as hopeful holobionts
Source: Microbiome. 2025 Jan 22;13:19. doi: 10.1186/s40168-024-01994-8 (PMC11752726; doi:10.1186/s40168-024-01994-8)
Supplement: Supplementary file 2 — Additional file 1. Supplementary analyses for microbiota diversity used in this study including figures 1.1-1.9. [file 40168_2024_1994_MOESM1_ESM.docx]

**Transgressive Hybrids as Hopeful Holobionts**

**Additional File 1: Additional Diversity Metrics**

**
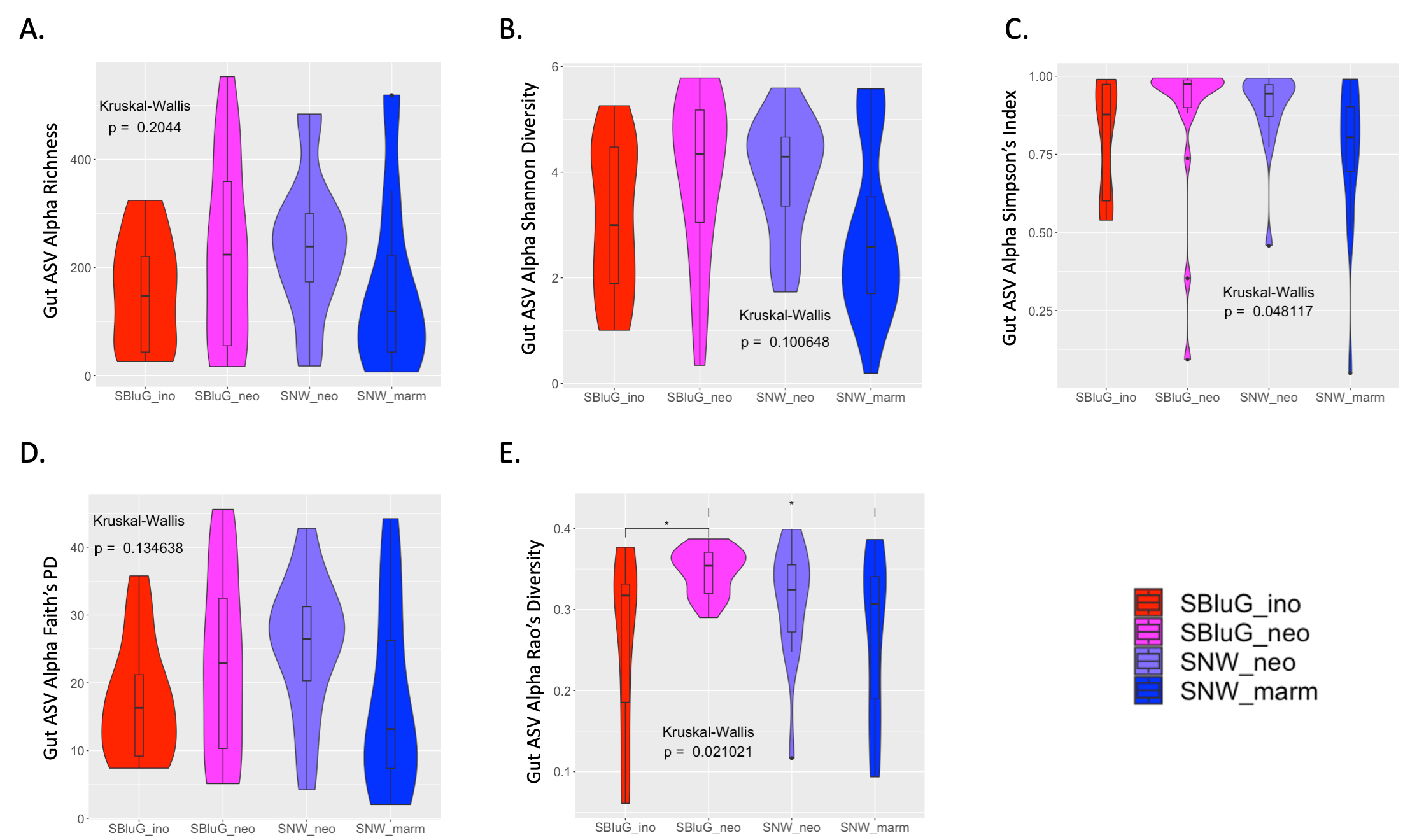
**

**Figure 1.1.** Comparison of gut microbiota 𝛼-diversity of amplicon sequence variants (ASVs) between populations of *Aspidoscelis inornatus* from SBluG (red), *A. neomexicanus* from SBluG (magenta), *A. neomexicanus* from SNW (purple), and *A. marmoratus* from SNW (blue) as measured by (A) richness (count of ASVs), (B) Shannon diversity, (C) Simpson’s index, (D) Faith’s phylogenetic diversity (PD), and (E) Rao’s diversity. Significant differences in diversity between groups, as determined by a Kruskal-Wallis test followed by post hoc pairwise Wilcox tests using a Benjamini-Hochberg correction, are indicated as follows: p-value ≤ 0.001 (***), p-value ≤ 0.01 (**), p-value ≤ 0.05 (*), p-value ≤ 0.1 (.).


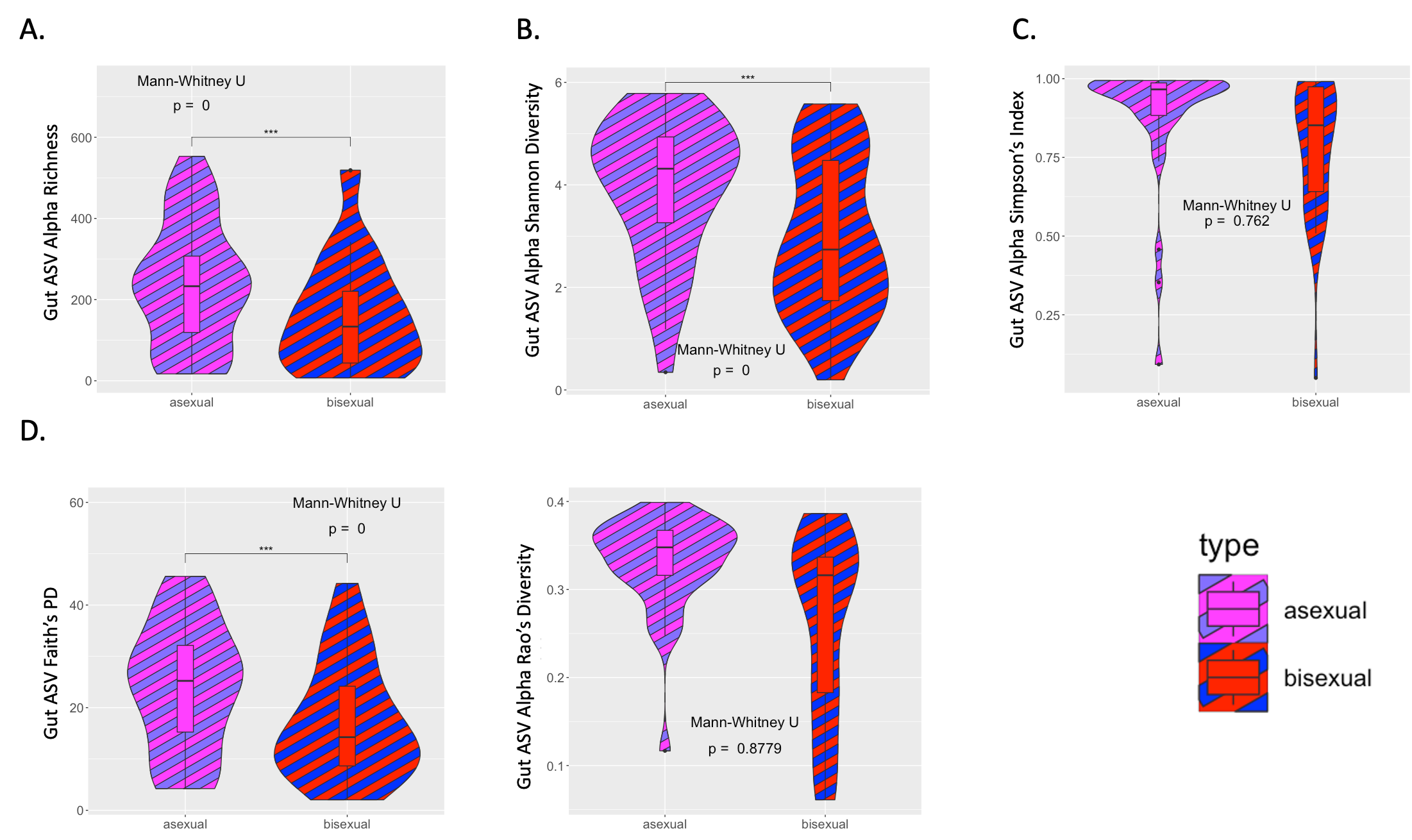


**Figure 1.2.** Comparison of gut microbiota 𝛼-diversity of amplicon sequence variants (ASVs) between parent sexual species (red and blue striped) and hybrid asexual *Aspidoscelis neomexicanus* (magenta and purple striped) as measured by (A) richness (count of ASVs), (B) Shannon diversity, (C) Simpson’s index, (D) Faith’s phylogenetic diversity (PD), and (E) Rao’s diversity. Significant differences in diversity between groups, as determined by a Mann-Whitney test, are indicated as follows: p-value ≤ 0.001 (***), p-value ≤ 0.01 (**), p-value ≤ 0.05 (*), p-value ≤ 0.1 (.).

**
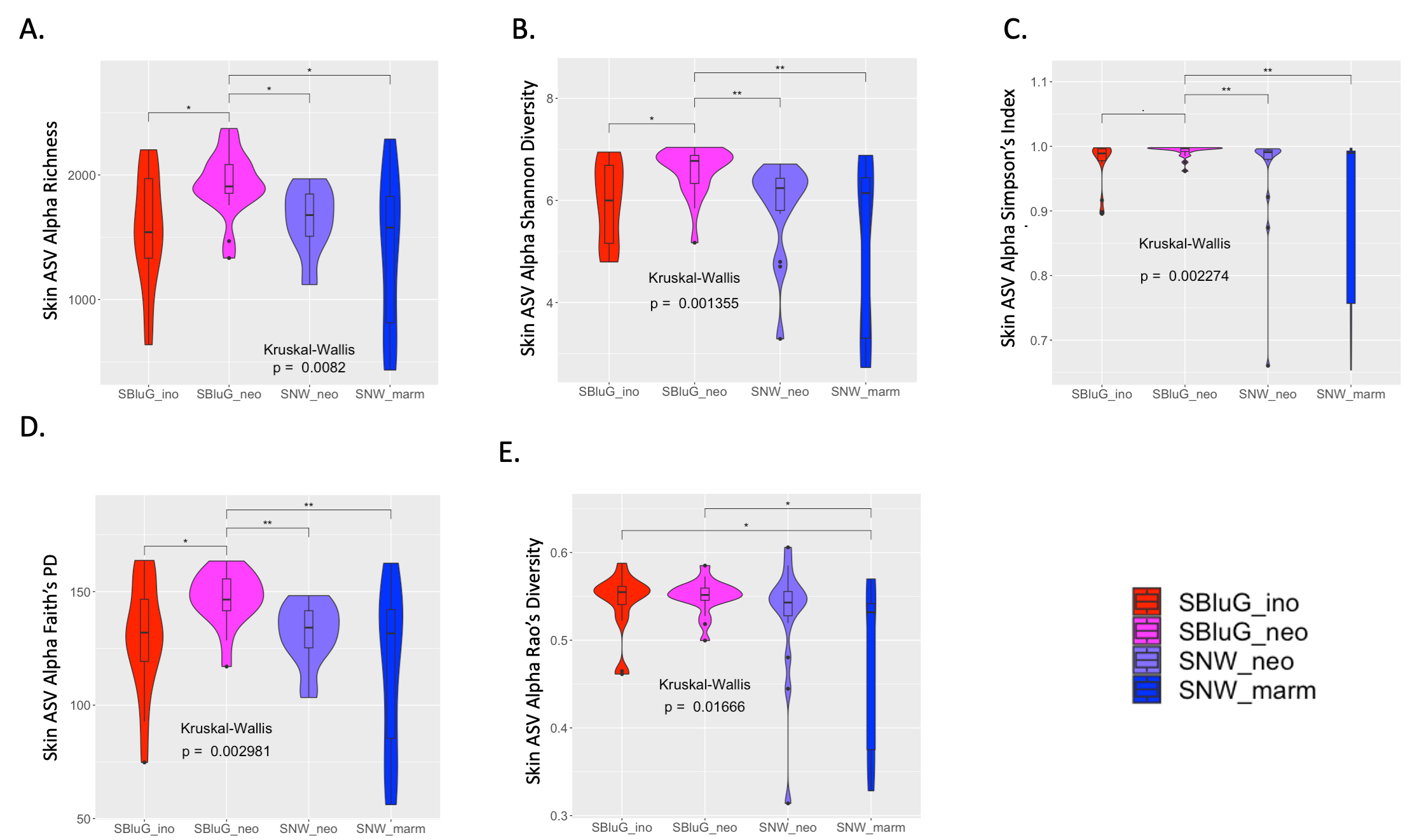
**

**Figure 1.3.** Comparison of skin microbiota 𝛼-diversity of amplicon sequence variants (ASVs) between populations of *Aspidoscelis inornatus* from SBluG (red), *A. neomexicanus* from SBluG (magenta), *A. neomexicanus* from SNW (purple), and *A. marmoratus* from SNW (blue) as measured by (A) richness (count of ASVs), (B) Shannon diversity, (C) Simpson’s index, (D) Faith’s phylogenetic diversity (PD), and (E) Rao’s diversity. Significant differences in diversity between groups, as determined by a Kruskal-Wallis test followed by post hoc pairwise Wilcox tests using a Benjamini-Hochberg correction, are indicated as follows: p-value ≤ 0.001 (***), p-value ≤ 0.01 (**), p-value ≤ 0.05 (*), p-value ≤ 0.1 (.).

**
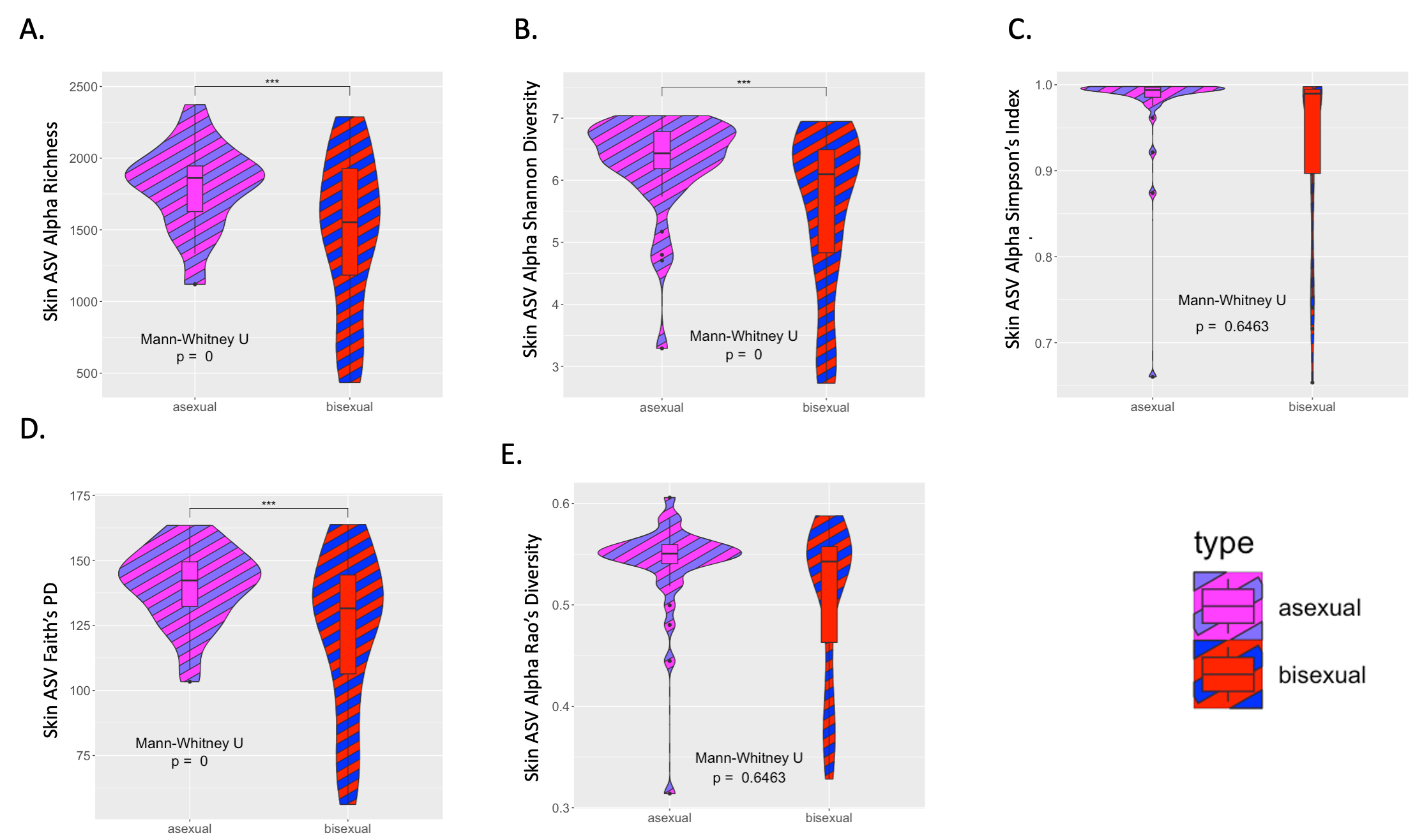
**

**Figure 1.4.** Comparison of skin microbiota 𝛼-diversity of amplicon sequence variants (ASVs) between parent sexual species (red and blue striped) and hybrid asexual *Aspidoscelis neomexicanus* (magenta and purple striped) as measured by (A) richness (count of ASVs), (B) Shannon diversity, (C) Simpson’s index, (D) Faith’s phylogenetic diversity (PD), and (E) Rao’s diversity. Significant differences in diversity between groups, as determined by a Mann-Whitney test, are indicated as follows: p-value ≤ 0.001 (***), p-value ≤ 0.01 (**), p-value ≤ 0.05 (*), p-value ≤ 0.1 (.).

**
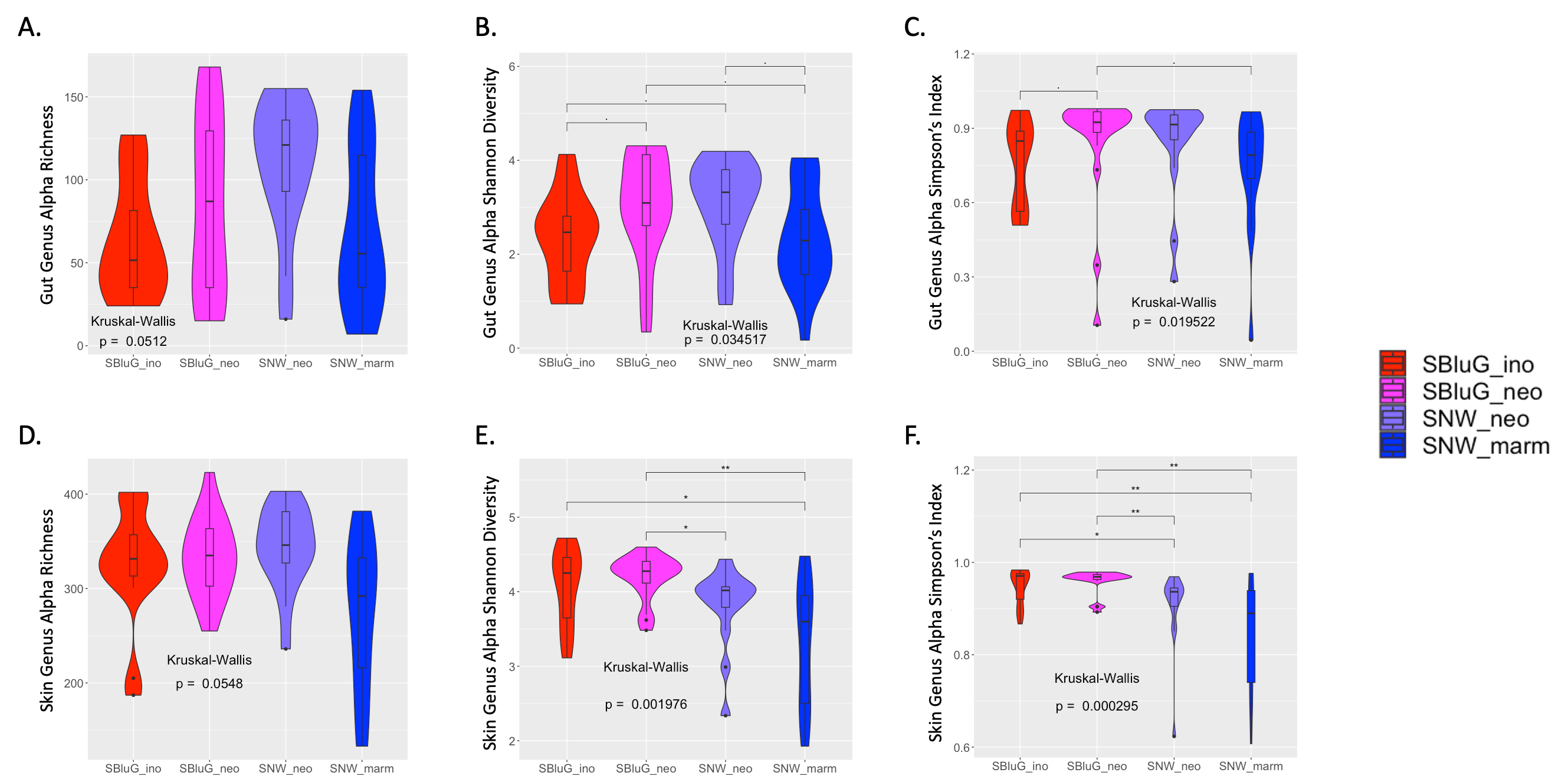
**

**Figure 1.5.** Comparison of gut (A-C) and skin (D-F) microbiota 𝛼-diversity of microbial genera between populations of *Aspidoscelis inornatus* from SBluG (red), *A. neomexicanus* from SBluG (magenta), *A. neomexicanus* from SNW (purple), and *A. marmoratus* from SNW (blue) as measured by (A, D) richness (count of genera), (B, E) Shannon diversity, and (C, F) Simpson’s index. Significant differences in diversity between groups, as determined by a Kruskal-Wallis test followed by post hoc pairwise Wilcox tests using a Benjamini-Hochberg correction, are indicated as follows: p-value ≤ 0.001 (***), p-value ≤ 0.01 (**), p-value ≤ 0.05 (*), p-value ≤ 0.1 (.).

**
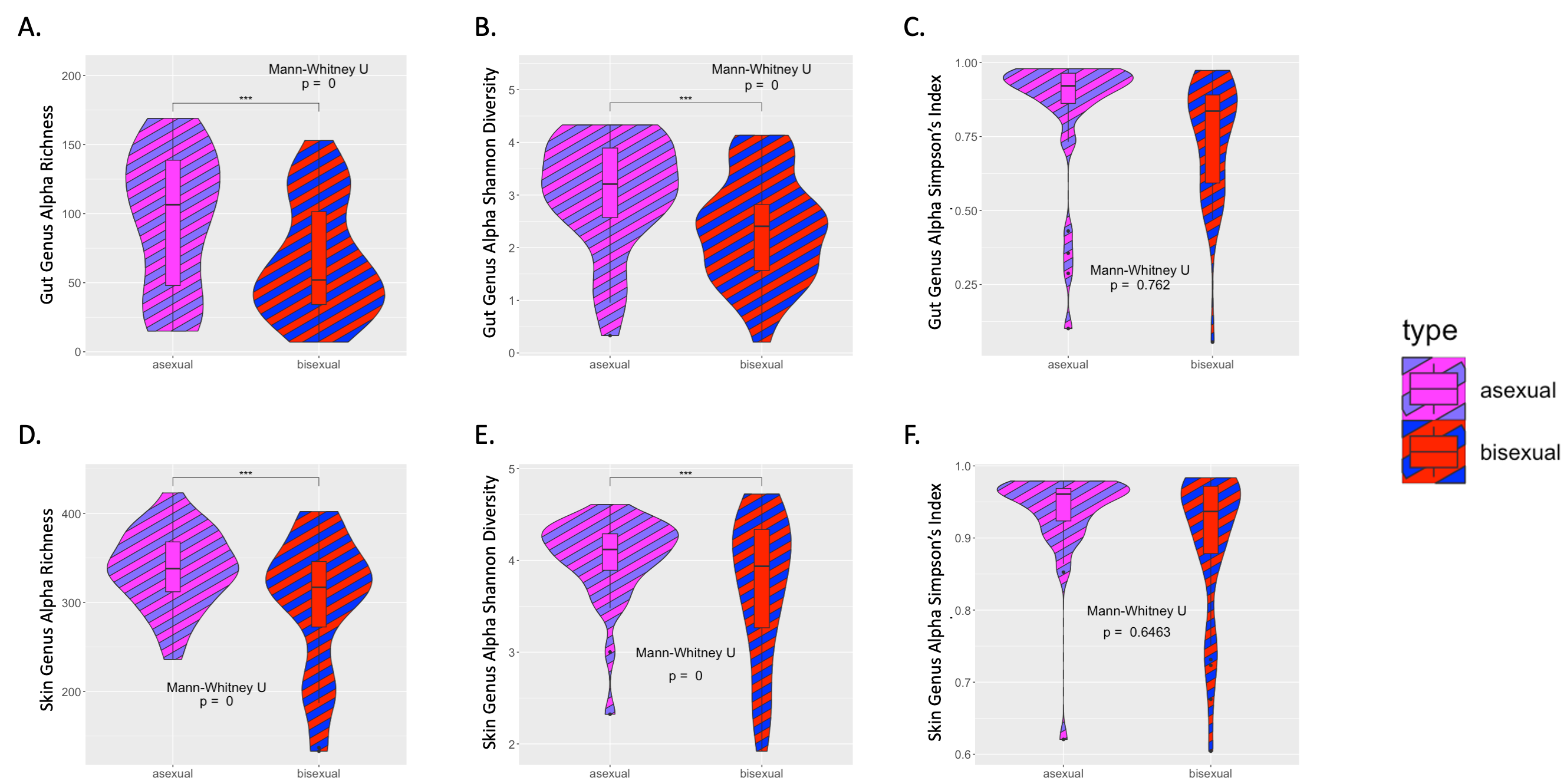
**

**Figure 1.6.** Comparison of gut (A-C) and skin (D-F) microbiota 𝛼-diversity of microbial genera between parent sexual species (red and blue striped) and hybrid asexual *Aspidoscelis neomexicanus* (magenta and purple striped) as measured by (A, D) richness (count of genera), (B, E) Shannon diversity, and (C, F) Simpson’s index. Significant differences in diversity between groups, as determined by a Mann-Whitney test, are indicated as follows: p-value ≤ 0.001 (***), p-value ≤ 0.01 (**), p-value ≤ 0.05 (*), p-value ≤ 0.1 (.).

**
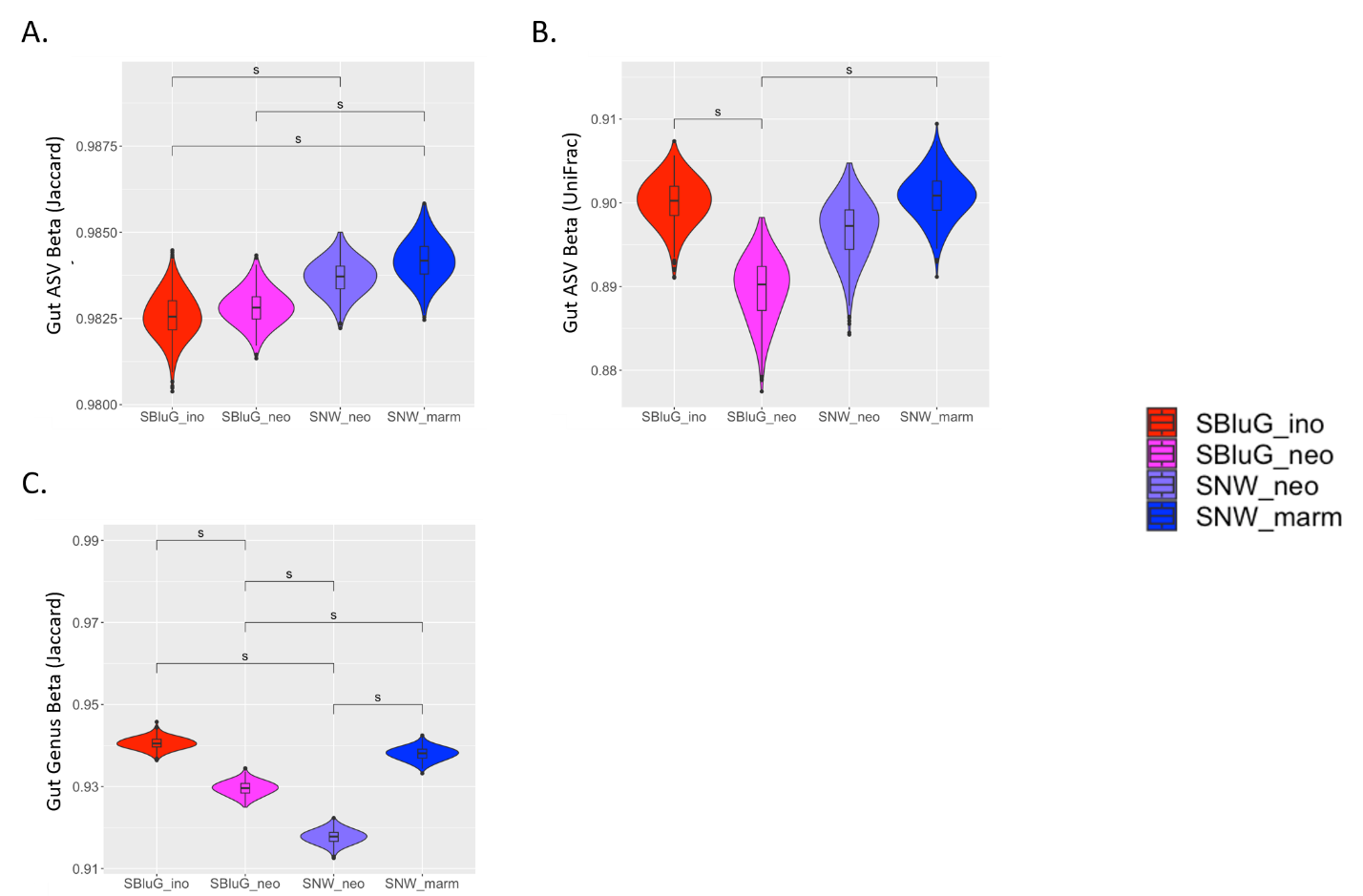
**

**Figure 1.7.** Comparison of gut microbiota 𝛽-diversity between populations of *Aspidoscelis inornatus* from SBluG (red), *A. neomexicanus* from SBluG (magenta), *A. neomexicanus* from SNW (purple), and *A. marmoratus* from SNW (blue) as measured using (A) Jaccard dissimilarity of amplicon sequence variants (ASVs), (B) unweighted UniFrac dissimilarity of ASVs, and (C) Jaccard dissimilarity of microbial genera. Each panel is based on 500 bootstraps of 15 lizards from each population using the methods for main Figure 1. Significant differences as determined by overlap of 83.4% confidence intervals are indicated with an ‘s.’

**
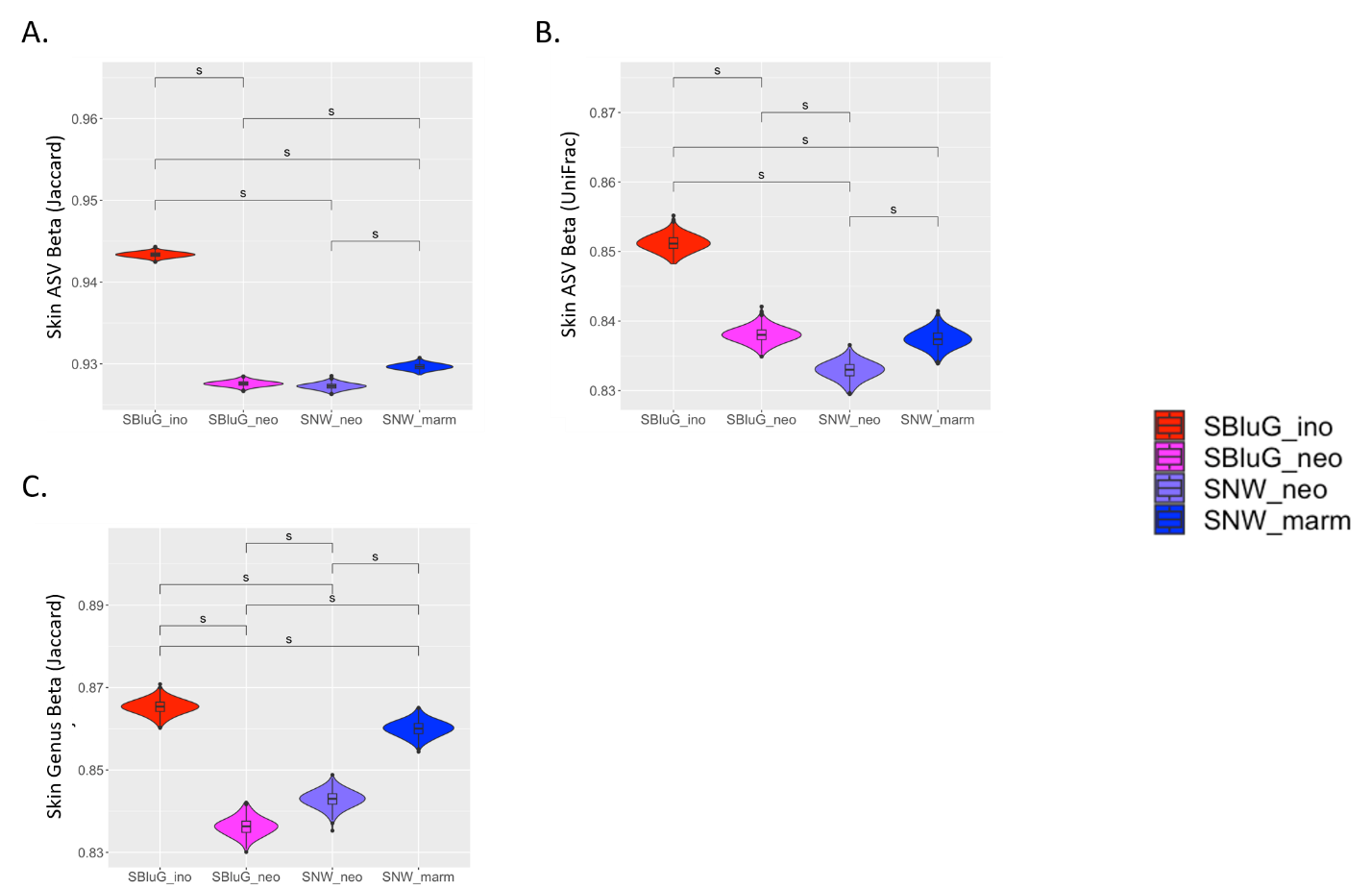
**

**Figure 1.8.** Comparison of skin microbiota 𝛽-diversity between populations of *Aspidoscelis inornatus* from SBluG (red), *A. neomexicanus* from SBluG (magenta), *A. neomexicanus* from SNW (purple), and *A. marmoratus* from SNW (blue) as measured using (A) Jaccard dissimilarity of amplicon sequence variants (ASVs), (B) unweighted UniFrac dissimilarity of ASVs, and (C) Jaccard dissimilarity of microbial genera. Each panel is based on 500 bootstraps of 15 lizards from each population using the methods for main Figure 1. Significant differences as determined by overlap of 83.4% confidence intervals are indicated with an ‘s.’


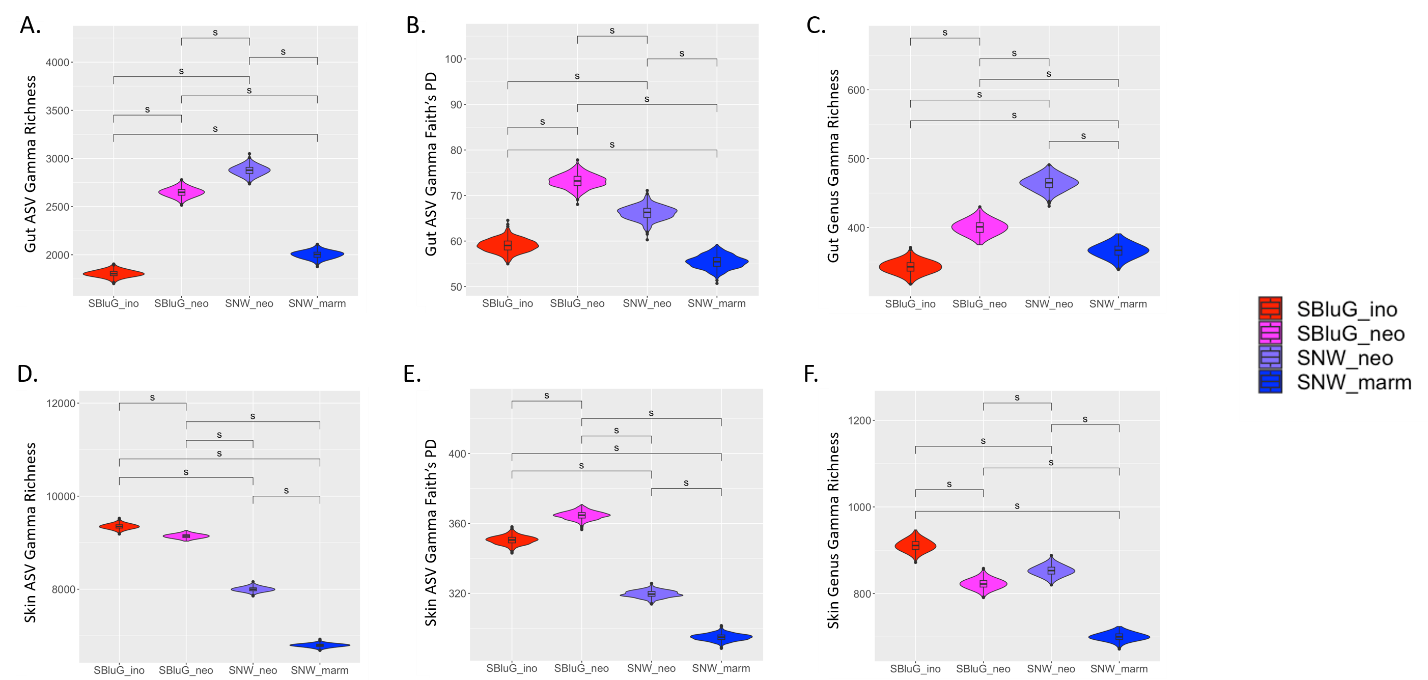


**Figure 1.9.** Comparison of gut (A-C) and skin (D-F) microbiota 𝛾-diversity between populations of *Aspidoscelis inornatus* from SBluG (red), *A. neomexicanus* from SBluG (magenta), *A. neomexicanus* from SNW (purple), and *A. marmoratus* from SNW (blue) as measured using (A, D) richness of amplicon sequence variants (ASV count), (B, E) Faith’s phylogenetic diversity (PD) of ASVs, and (C, F) richness of microbial genera (genera count). Each panel is based on 500 bootstraps of 15 lizards from each population using the methods for main Figure 1. Significant differences as determined by overlap of 83.4% confidence intervals are indicated with an ‘s.’
