# Supplementary material for: Transgressive hybrids as hopeful holobionts
Source: Microbiome. 2025 Jan 22;13:19. doi: 10.1186/s40168-024-01994-8 (PMC11752726; doi:10.1186/s40168-024-01994-8)
Supplement: Supplementary file 3 — Additional file 2. Supplementary analyses for core microbiota diversity used in this study including figures 2.1-2.5. [file 40168_2024_1994_MOESM2_ESM.docx]

**Transgressive Hybrids as Hopeful Holobionts**

**Additional File 2: Core Microbiota Analyses**

**
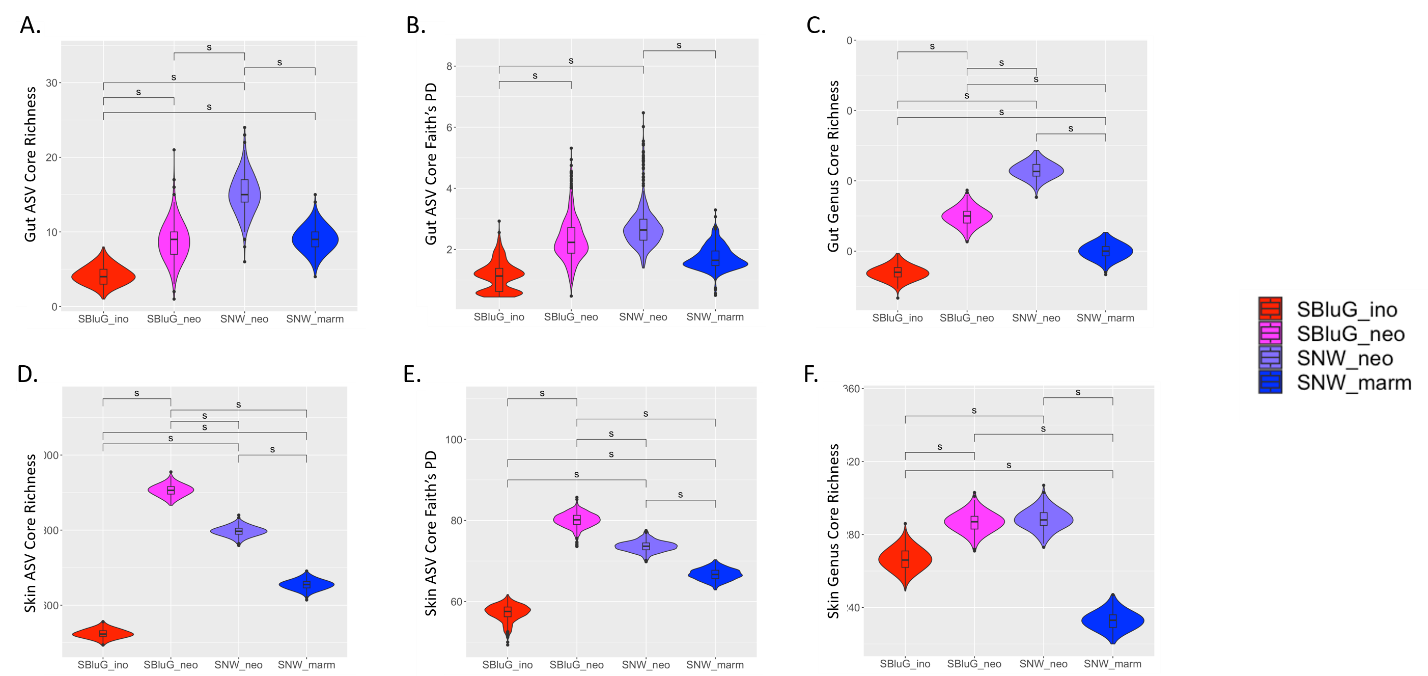
**

**Figure 2.1.** Comparison of the diversity of the core gut (A-C) and skin (D-F) microbiota between populations of *Aspidoscelis inornatus* from SBluG (red), *A. neomexicanus* from SBluG (magenta), *A. neomexicanus* from SNW (purple), and *A. marmoratus* from SNW (blue) as measured using (A, D) richness of amplicon sequence variants (ASV count), (B, E) Faith’s phylogenetic diversity (PD) of ASVs, and (C, F) richness of microbial genera (genera count). Each panel is based on 500 bootstraps of 15 lizards from each population using the methods for main Figure 2. Significant differences as determined by overlap of 83.4% confidence intervals are indicated with an ‘s.’

**
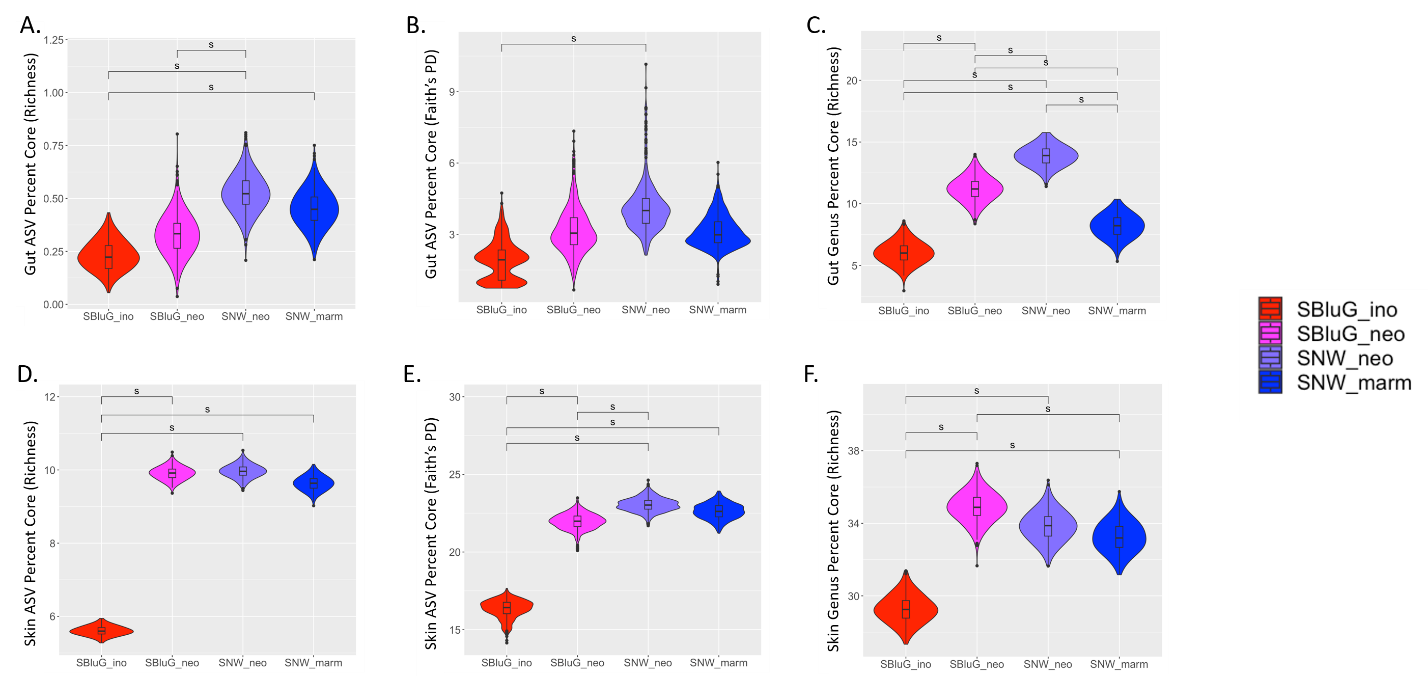
**

**Figure 2.2.** Comparison of the percentage of the microbiota that is part of the core for gut (A-C) and skin (D-F) microbiota between populations of *Aspidoscelis inornatus* from SBluG (red), *A. neomexicanus* from SBluG (magenta), *A. neomexicanus* from SNW (purple), and *A. marmoratus* from SNW (blue) as measured using (A, D) richness of amplicon sequence variants (ASV count), (B, E) Faith’s phylogenetic diversity (PD) of ASVs, and (C, F) richness of microbial genera (genera count). Each panel is based on 500 bootstraps of 15 lizards from each population using the methods for main Figure 2. Significant differences as determined by overlap of 83.4% confidence intervals are indicated with an ‘s.’


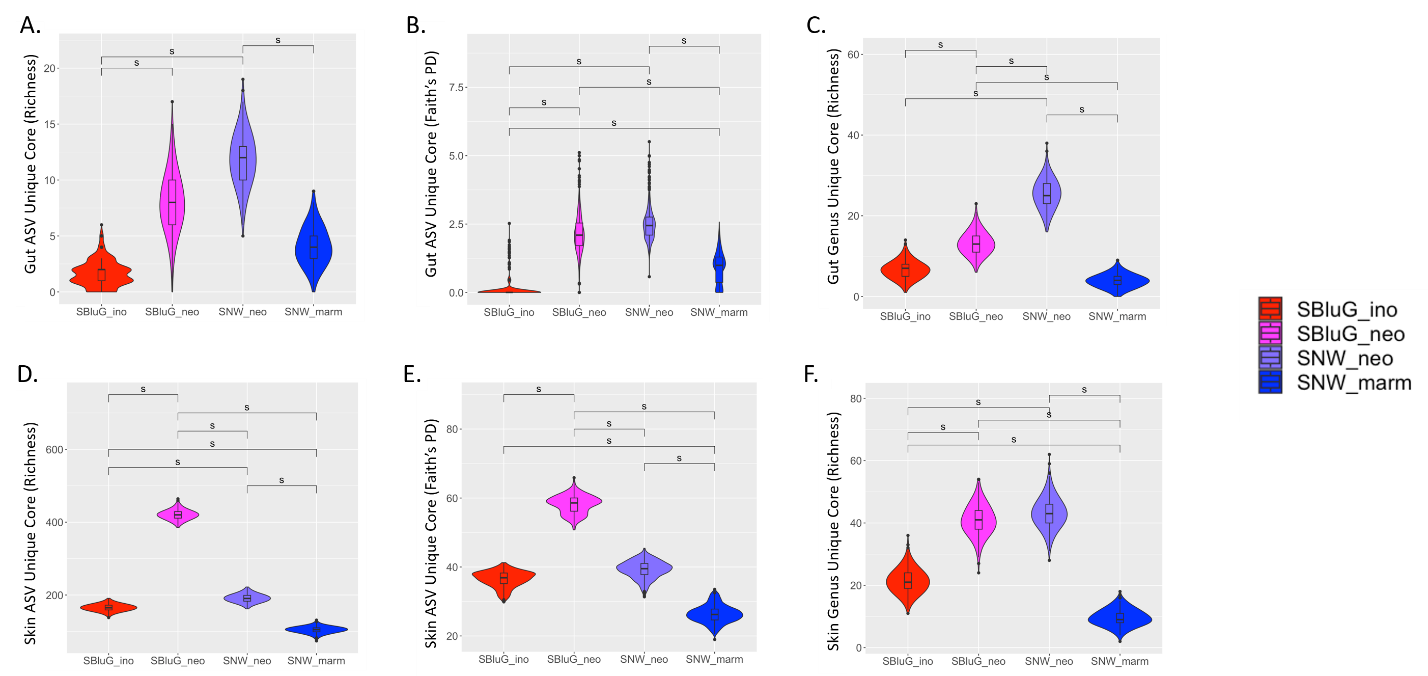


**Figure 2.3.** Comparison of the diversity of the unique component of the core gut (A-C) and skin (D-F) microbiota of populations of *Aspidoscelis inornatus* from SBluG (red), *A. neomexicanus* from SBluG (magenta), *A. neomexicanus* from SNW (purple), and *A. marmoratus* from SNW (blue) as measured using (A, D) richness of amplicon sequence variants (ASV count), (B, E) Faith’s phylogenetic diversity (PD) of ASVs, and (C, F) richness of microbial genera (genera count). Each panel is based on 500 bootstraps of 15 lizards from each population using the methods for main Figure 2. Significant differences as determined by overlap of 83.4% confidence intervals are indicated with an ‘s.’


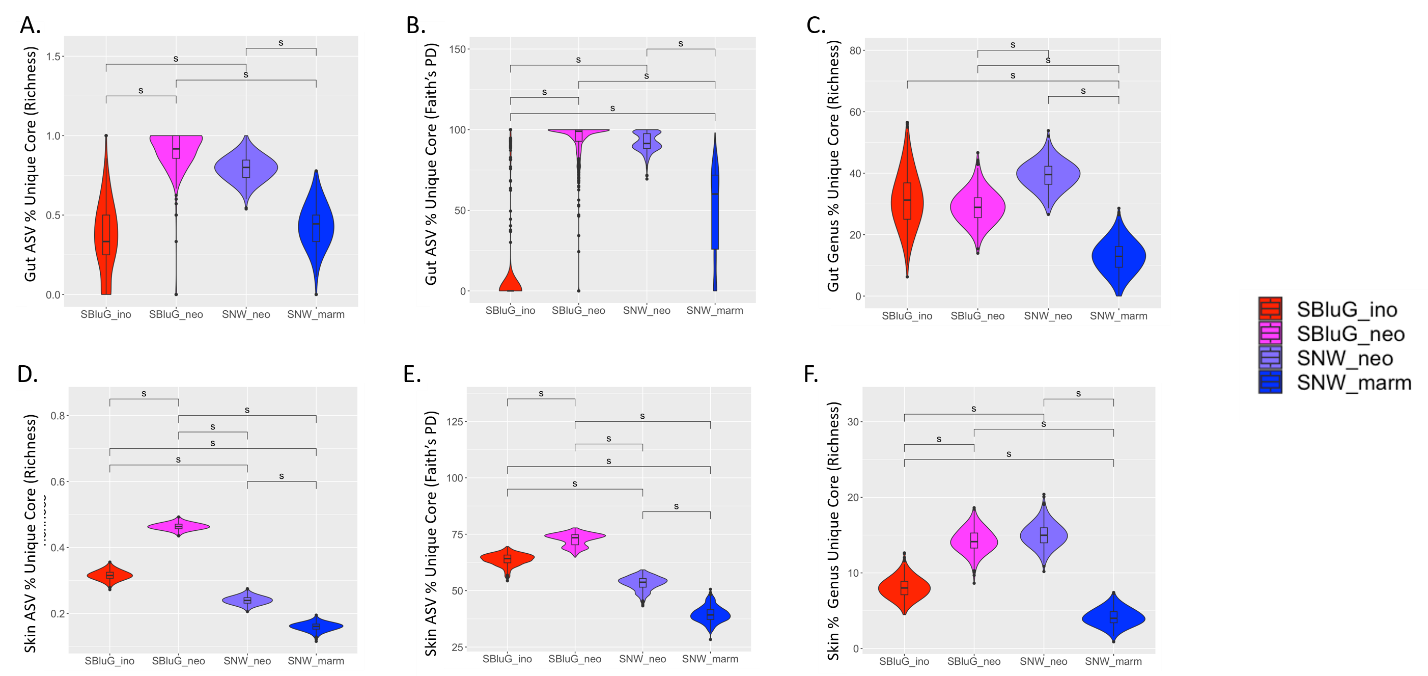


**Figure 2.4.** Comparison of the percentage of the core microbiota that is unique to a particular population for gut (A-C) and skin (D-F) microbiota between populations of *Aspidoscelis inornatus* from SBluG (red), *A. neomexicanus* from SBluG (magenta), *A. neomexicanus* from SNW (purple), and *A. marmoratus* from SNW (blue) as measured using (A, D) richness of amplicon sequence variants (ASV count), (B, E) Faith’s phylogenetic diversity (PD) of ASVs, and (C, F) richness of microbial genera (genera count). Each panel is based on 500 bootstraps of 15 lizards from each population using the methods for main Figure 2. Significant differences as determined by overlap of 83.4% confidence intervals are indicated with an ‘s.’


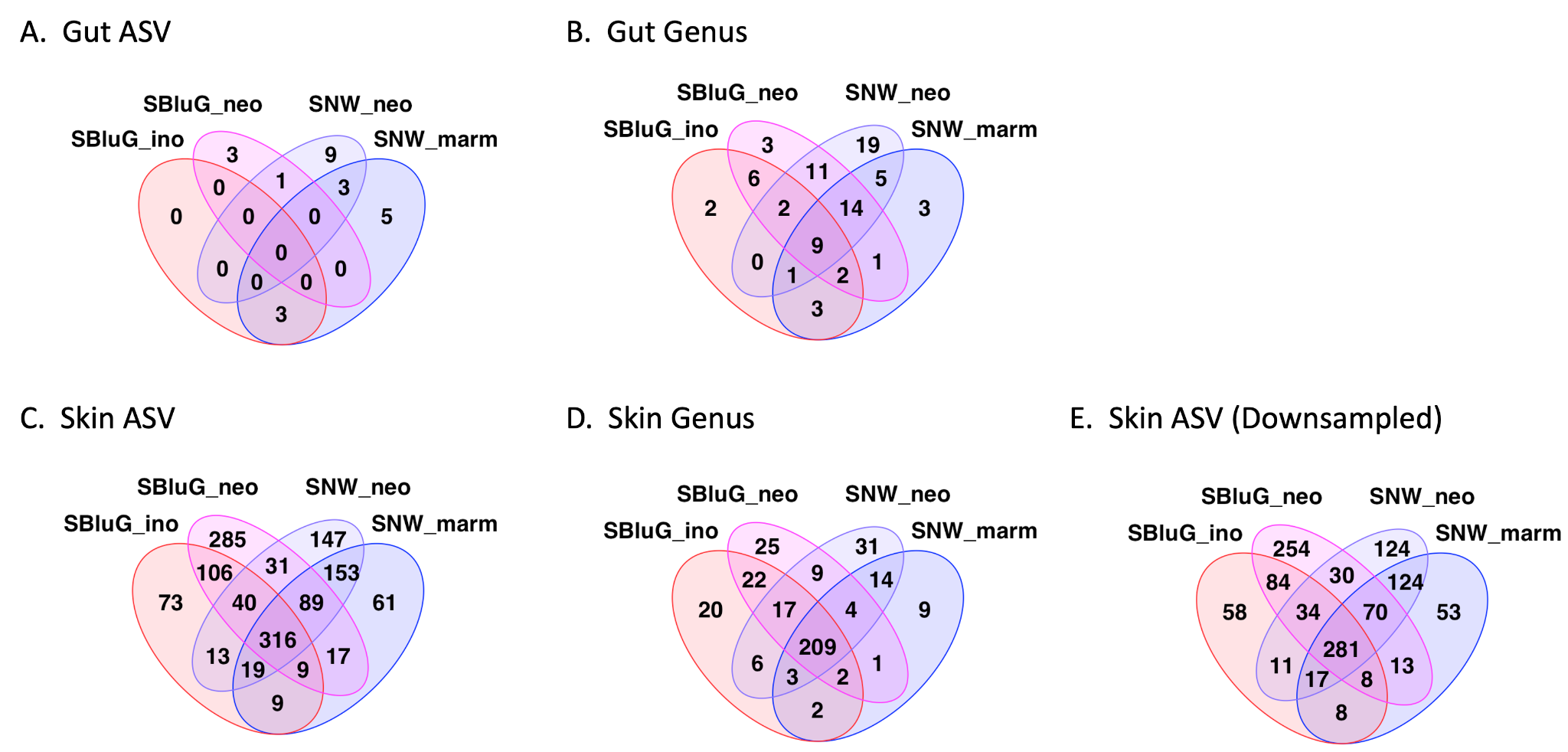


**Figure 2.5.** Venn diagrams showing the number of amplicon sequence variants (ASVs; A, C, E) and genera (B, D) shared among gut (A, B) and skin (C-E) microbiota of *Aspidoscelis inornatus* at SBluG (red), *A. marmoratus* at SNW (blue), *A. neomexicanus* at SBluG (magenta), and *A. neomexicanus* at SNW (purple). For all panels, a microbial ASV or genus is considered part of the core microbiota if it is found on at least 50% of individual lizards from a population. For panels A-D, microbiota were rarefied to the minimum reads present from the lizard microbiota with the lowest read count (gut: 11412 reads, skin: 116931). For panel E, we rarefied the skin microbiota to 11,412 reads, consistent with the depth available from our gut microbiota samples.
