# Supplementary material for: Transgressive hybrids as hopeful holobionts
Source: Microbiome. 2025 Jan 22;13:19. doi: 10.1186/s40168-024-01994-8 (PMC11752726; doi:10.1186/s40168-024-01994-8)
Supplement: Supplementary file 11 — Additional file 10. Supplementary information for lizard marking method implemented in this study including figure 10.1. [file 40168_2024_1994_MOESM10_ESM.docx]

**Transgressive Hybrids as Hopeful Holobionts**

**Additional file 10: Marking *Aspidoscelis* Lizards**


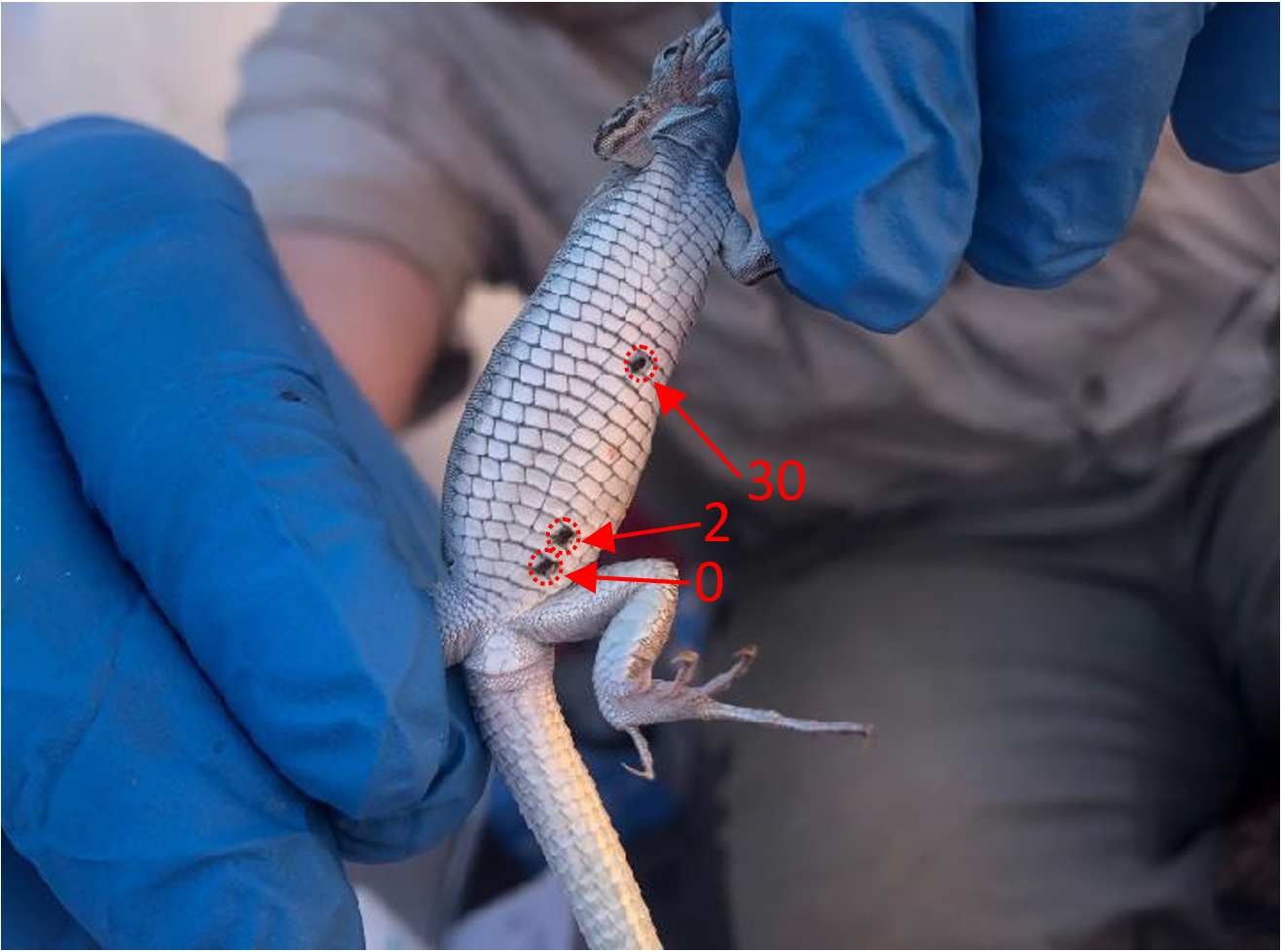


**Figure 10.1.** Ventral aspect of adult *Aspidoscelis neomexicanus* (#32) marked with a miniature medical cautery unit. The marking scheme consists of marking individuals on the same ventral scale column (along the anterior-posterior axis). The most posterior mark near the left hind-leg indicates ‘0’ and is used to calibrate the marks positioned anteriorly. Scale-by-scale, counting anterior to the calibration mark, scales indicated 1-10 by increments of one. At the 11^th^ scale anterior to the calibration mark, each scale indicates increments of 10 (i.e., 20, 30, 40, 50,… n).
